# Peptimapper: proteogenomics workflow for the expert annotation of eukaryotic genomes

**DOI:** 10.1186/s12864-019-5431-9

**Published:** 2019-01-17

**Authors:** Laetitia Guillot, Ludovic Delage, Alain Viari, Yves Vandenbrouck, Emmanuelle Com, Andrés Ritter, Régis Lavigne, Dominique Marie, Pierre Peterlongo, Philippe Potin, Charles Pineau

**Affiliations:** 1 Univ Rennes, Inserm, EHESP, Irset (Institut de recherche en santé, environnement et travail) – UMR_S 1085, F-35042 Rennes cedex, France; 2 Sorbonne Université, UPMC, CNRS, UMR 8227, Integrative Biology of Marine Models, Biological Station, CS 90074, F-29688 Roscoff, France; 3 INRIA Grenoble-Rhône-Alpes, F-38330 Montbonnot-Saint-Martin, France; 4 University Grenoble Alpes, CEA, Inserm, BIG-BGE, 38000 Grenoble, France; 5 Present address: Sorbonne Université, CNRS, Institut de Biologie Paris-Seine, Laboratory of Computational and Quantitative Biology, F-75005 Paris, France; 6 University Rennes, Inria, CNRS, IRISA, F-35042 Rennes, France; 7 Protim, Univ Rennes, F-35042 Rennes cedex, France

**Keywords:** Bioinformatics, Genome annotation, Peptide sequence tag, Proteogenomics, Proteomics, Tandem mass spectrometry

## Abstract

**Background:**

Accurate structural annotation of genomes is still a challenge, despite the progress made over the past decade. The prediction of gene structure remains difficult, especially for eukaryotic species, and is often erroneous and incomplete. We used a proteogenomics strategy, taking advantage of the combination of proteomics datasets and bioinformatics tools, to identify novel protein coding-genes and splice isoforms, assign correct start sites, and validate predicted exons and genes.

**Results:**

Our proteogenomics workflow, Peptimapper, was applied to the genome annotation of *Ectocarpus sp.*, a key reference genome for both the brown algal lineage and stramenopiles. We generated proteomics data from various life cycle stages of *Ectocarpus sp.* strains and sub-cellular fractions using a shotgun approach. First, we directly generated peptide sequence tags (PSTs) from the proteomics data. Second, we mapped PSTs onto the translated genomic sequence. Closely located hits (i.e.*,* PSTs locations on the genome) were then clustered to detect potential coding regions based on parameters optimized for the organism. Third, we evaluated each cluster and compared it to gene predictions from existing conventional genome annotation approaches. Finally, we integrated cluster locations into GFF files to use a genome viewer. We identified two potential novel genes, a ribosomal protein L22 and an aryl sulfotransferase and corrected the gene structure of a dihydrolipoamide acetyltransferase. We experimentally validated the results by RT-PCR and using transcriptomics data.

**Conclusions:**

Peptimapper is a complementary tool for the expert annotation of genomes. It is suitable for any organism and is distributed through a Docker image available on two public bioinformatics docker repositories: Docker Hub and BioShaDock. This workflow is also accessible through the Galaxy framework and for use by non-computer scientists at https://galaxy.protim.eu.

Data are available via ProteomeXchange under identifier PXD010618.

**Electronic supplementary material:**

The online version of this article (10.1186/s12864-019-5431-9) contains supplementary material, which is available to authorized users.

## Background

Proteomics and genomics data combined with bioinformatics tools, known as proteogenomics [1–3], is a valuable strategy to improve genome annotation [4–6]. Proteomics methods and applications have been reviewed by Nesvizhskii [7] and more recently by Menschaert & Fenyö and by Ruggles and collaborators [8, 9]. Proteomics data provides direct access to amino-acid sequences that can be mapped onto translated genomic sequences [10, 11]. The combined use of experimental proteomics data and genomic sequences is a powerful way to: i) confirm gene-model predictions, ii) correct possible intron/exon boundary errors or wrong start/stop codons, and iii) find new CDSs that have not been computationally predicted by machine learning-based approaches or homology searches. Many studies have demonstrated the use proteomics datasets to provide protein-level evidence of gene expression and refine gene models [3, 12]. This approach has been successfully applied to many organisms, such as *Anopheles gambiae* [13], *Rattus norvegicus* [14, 15], and *Homo sapiens* [16]*,* as well as plants [17–19]. Many microbial genomes, usually lacking high quality annotation, can also benefit from proteogenomics strategies to improve gene prediction [20–24]. Finally, proteogenomics can also significantly influence the study of non-model organisms [25].

**Fig. 1:**
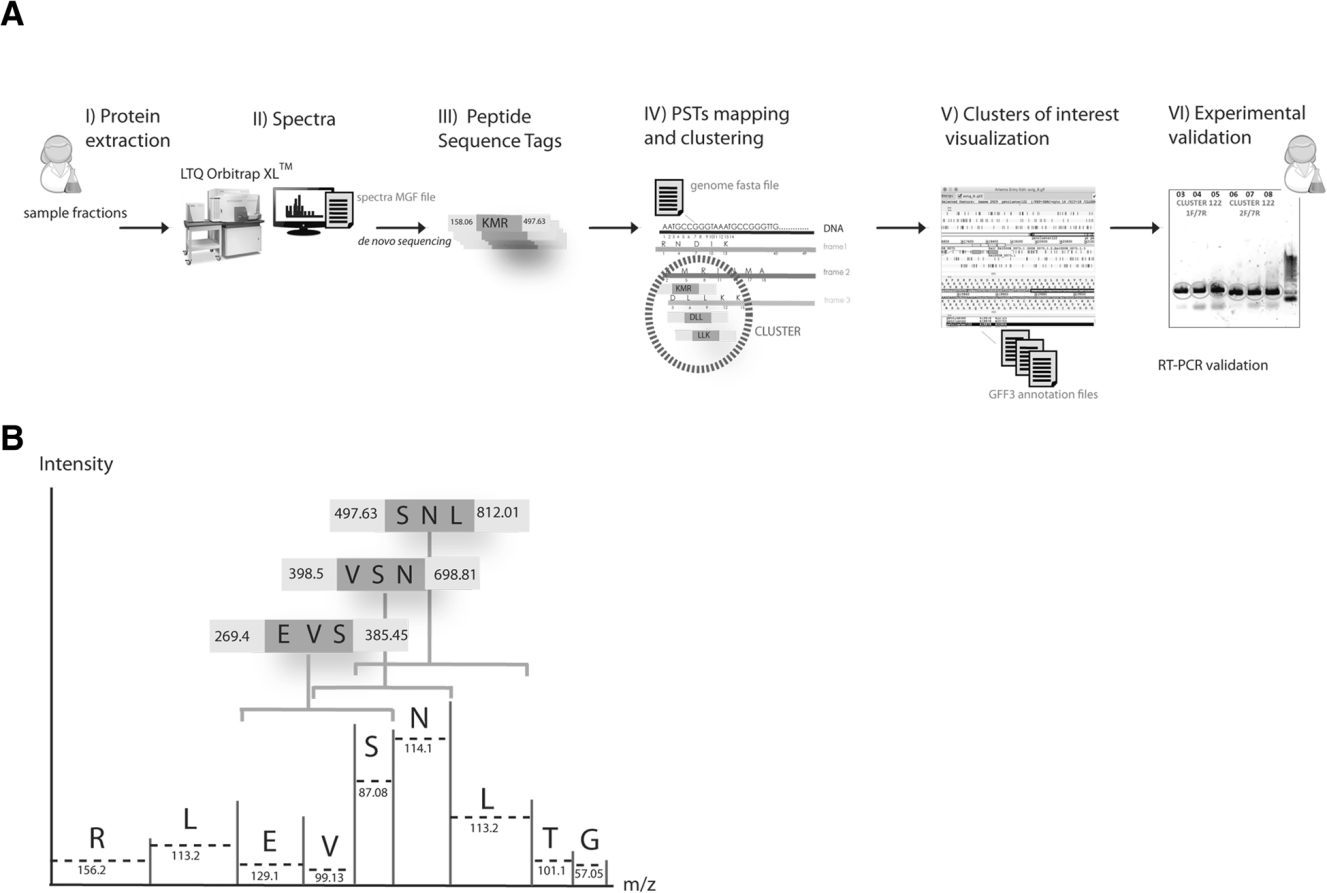
**a** Project workflow: Samples corresponding to various stages of the life cycle (sporophyte, gametophyte, and gametes) and sub-cellular compartments of *Ectocarpus sp.* were prepared for MS analysis. PSTs were generated from the MS/MS data, mapped against the genome, and clustered. We classified each cluster according to their genomic annotation (whether one hit overlapped with or included at least one CDS of an identified protein) and the number of typical spectra and verified whether all hits were included in a CDS or not. The location of clusters of interest were written into GFF files to be integrated into a Genome viewer. The results of cluster qualification and visualization along the contig allowed us to select clusters for experimental characterization. **b** Illustration of PSTs obtained from an MS/MS spectrum. MS/MS spectra are composed of ions resulting from the fragmentation of peptides at their peptide bonds during tandem mass spectrometry analysis. The generated fragment ions differ in mass corresponding to their adjacent amino-acid masses within the peptide sequence. A PST is a partial element of information deduced from an MS/MS spectrum, defined as a small sequence of several probable adjacent amino acids from the original peptide and the masses of the flanking N- and C-terminal fragment ions of this small sequence

Here we developed an easy to use, suitable, and efficient proteogenomics workflow, Peptimapper (Fig. 1a), to complete eukaryotic genome annotation. It automatically generates de novo short amino-acid sequences (i.e., peptide sequence tags, PSTs) from experimental proteomics data, maps these to the six-frame translation of genomics DNA sequences, and highlights potentially translated regions, which could be exons or genes. Our workflow makes it possible to not only improve genome annotation by confirming or correcting gene models or finding new CDSs, but also to complete classical database-driven proteomics identification, by generating a list of gene-matched translated proteins using these short de novo amino-acid sequences.

## Design and implementation

Our proteogenomics workflow (Fig. 1a), Peptimapper, is composed of a series of scripts we developed for a project called *Ectoline.* The scripts are partially based on the PepLine software [18], which were tested and some modified. The workflow consists of modular components and can therefore be used for any eukaryotic genome, following modification to accommodate its properties. We developed Peptimapper using the genome of *Ectocarpus sp.,* a key reference genome for both the brown algal lineage and stramenopiles. For each biological sample, we generated the MS/MS spectra file in Mascot Generic File (MGF) format using conventional proteomics software (i.e.*, Mascot Distiller*, *Proteome Discoverer™*, etc.). We used the “sequence tagging” approach [10], in which a PST is defined by a small sequence tag (usually three or four amino acids) and the two flanking (N- and C-terminal) masses (Fig. 1b).

**Fig. 2:**
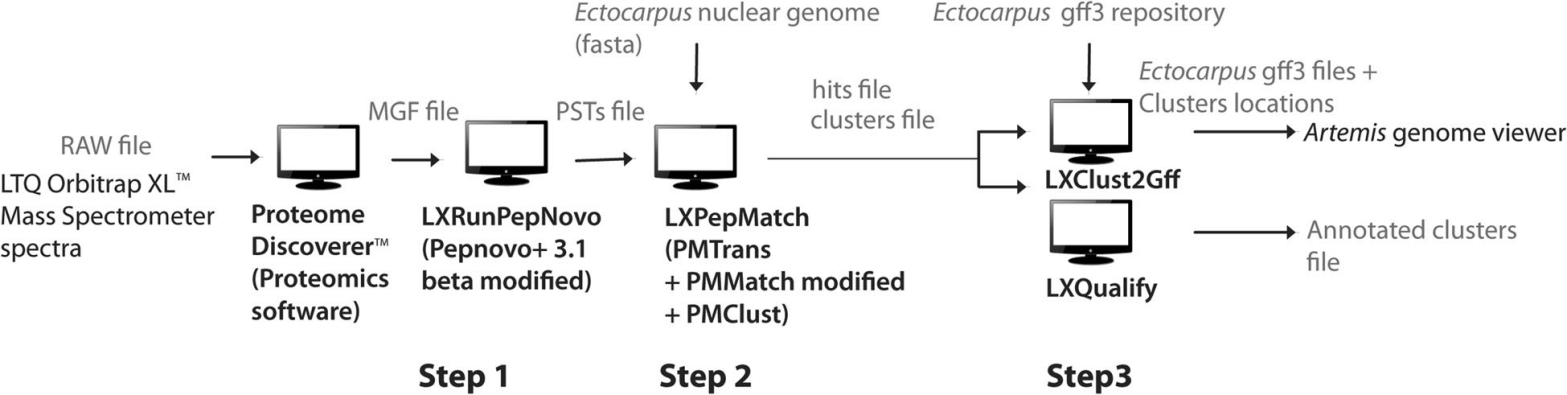
Steps of the bioinformatics workflow

The bioinformatics steps are shown in Fig. 2. PSTs were generated de novo from the MS/MS spectra information of the MGF. After testing several PSTs generation tools (see Step 1: From MGF files to PSTs), we decided to adapt an existing tool: *PepNovo +* 3.1 beta [26] (*LXRunPepNovo*). PSTs were then mapped on the six-frame translations of the genome sequence, resulting in a list of hits. A hit is defined as the location of a PST on the genome sequence. Finally, closely located hits were clustered to identify regions potentially associated with genes or, at least exons. This was achieved by testing (see Step 2: PST mapping and clustering below) and bundling three modules of the PepLine software (*PMTrans, PMMatch*, *PMClust*) into one script*: LXPepMatch.*

By cross-checking results using a classical database-driven proteomics approach, we tested and optimized step 1 and step 2 modules by varying settings according to known *Ectocarpus sp.* genome features (downloaded from ORCAE, a public database: http://bioinformatics.psb.ugent.be/orcae/overview/Ectsi) and using as input a subset of reference spectra (see MS/MS reference datasets building). The workflow construction is detailed below (see section Workflow construction: step-by-step).

The last step consisted of classifying clusters generated in step 2, according to their annotation and confidence level using the script we developed for this purpose: *LXQualify*. Clusters on the genome were visualized by implementing another specific script, *LXClust2Gff*. It wrote cluster locations generated by *LXPepMatch* into GFF files for further integration into a genome viewer (i.e., *Artemis* (Sanger Institute, England) [27]). Results were validated by manually comparing various clusters of interest with EST and RNA-Seq data (see workflow building step by step, step 3 section, below).

### From biological samples to MS/MS spectra files (MGF files)

We extracted various subcellular samples (cell walls, cytoplasm, nuclei, membranes) from various life cycle stages of *Ectocarpus sp.* strains (gametophyte, gamete) using specific protocols (see Additional file 1) to provide deep coverage of the proteome. Enriched extracts were then separated by SDS-PAGE onto 12% precast GeBaGels (Gene Bio-Application Ltd., Kfar Hanagide, Israel) and stained with EZBlue gel staining reagent (Sigma-Aldrich, Saint-Quentin Fallavier, France), according to the manufacturer’s instructions. Gel lanes were cut into 20 bands which were subjected to trypsin digestion, as previously described [28]. Tryptic peptides were analyzed using a nanoflow high-performance liquid chromatography (HPLC) system (LC Packings Ultimate 3000, Thermo Fisher Scientific, Courtaboeuf, France) connected to a hybrid LTQ-Orbitrap XL™ spectrometer (Thermo Fisher Scientific) equipped with a nanoelectrospray ion source (New Objective, Woburn, Massachusetts, USA), as previously described [29]. The mass spectrometer was operated in the data-dependent mode by automatic switching between full-survey scan MS and consecutive MS/MS acquisition. Survey full scan MS spectra (mass range 400–2000) were acquired in the Orbitrap section of the instrument with a resolution of *r* = 60,000 at 400 m/z; ion injection times were calculated for each spectrum to allow the accumulation of 10^6^ ions in the Orbitrap. The seven most intense peptide ions in each survey scan with an intensity above 2000 were sequentially isolated and fragmented in the linear ion trap by collision-induced dissociation. For Orbitrap measurements, an external calibration was used before each injection series to ensure an overall error mass accuracy below 5 ppm for the detected peptides. MS data were saved in RAW file format (Thermo Fisher Scientific) using XCalibur 2.0.7 with tune 2.4. For each sample, MS/MS spectra, grouped into an MGF file, were generated by *Proteome Discoverer™* 1.2 software. The mass spectrometry proteomics data have been deposited to the ProteomeXchange Consortium via the PRIDE [30] partner repository with the dataset identifier PXD010618.

### Building the MS/MS reference dataset

For each step of Peptimapper, we developed specific tools or used or adapted existing tools. We built three reference datasets for their assessment. These were obtained by a classical database-driven proteomics approach. Peptides were identified using *Proteome Discoverer™* 1.2 software supported by the *Mascot* search engine (Mascot server v2.2.07; http://www.matrixscience.com), using its decoy strategy. This software matches each MS/MS experimental spectrum (RAW file) against a database comprising all theoretical MS/MS spectra calculated for every possible peptide from an in silico digestion of *Ectocarpus sp.* gene model proteins (downloaded from ORCAE, https://bioinformatics.psb.ugent.be/gdb/ectocarpus/, Ectsi_prot, 2010, 16,533 sequences). Mass tolerance was set to 10 ppm and 0.5 Da for MS and MS/MS, respectively. Enzyme selectivity was set to full trypsin, with one missed cleavage allowed. The allowed protein modifications were set to carbamidomethylation of cysteines and variable oxidation of methionine. *Proteome Discoverer™* identification results allowed us to manually create reference MGF files composed of MS/MS spectra, selected according to the False Discovery Rate (FDR) calculated in *Proteome Discoverer™* and the reliability of the protein identification. MGF files were separated into three reference datasets according to the confidence level of the identifications: the “*green*” reference dataset (high quality spectra) containing all MS/MS spectra corresponding to identified proteins with at least three peptides and a FDR (computed as described above by the Mascot search engine) < 1%; the “*orange*” reference dataset (medium quality spectra) containing all MS/MS spectra with proteins identified by one peptide or more, and a FDR > 1% and < 5% and; the “*red*” reference dataset (low quality spectra) containing all MS/MS spectra without any protein identification and a FDR > 5%.

### Workflow construction: step-by-step

#### Step 1: From MGF files to PSTs

The first step (Fig. 2) consisted of PST generation. We considered three bioinformatics tools for this step: 1) *PepNovo + 3.1* [26]*,* 2) *Peaks* [31]*,* and 3) *Taggor,* which is a module of PepLine [18]*. Taggor,* initially developed for QTOF mass spectrometer data treatment, was adapted to account for mass tolerance parameters when using an ion trap spectrometer. We tested tool performance using a subset of the *green* dataset: 10 high quality MS/MS spectra provided by *proteome Discoverer™*, exporting the spectra of the two best peptides from each of the five top scoring proteins (see Additional file 2) into a MGF file. This file was used as input for each of the three bioinformatics tools. We then selected the one that generated the most PSTs identical to peptide sequences identified by *Proteome Discoverer™* for the same spectra.

#### Step 2: PSTs mapping and clustering

**Fig. 3:**
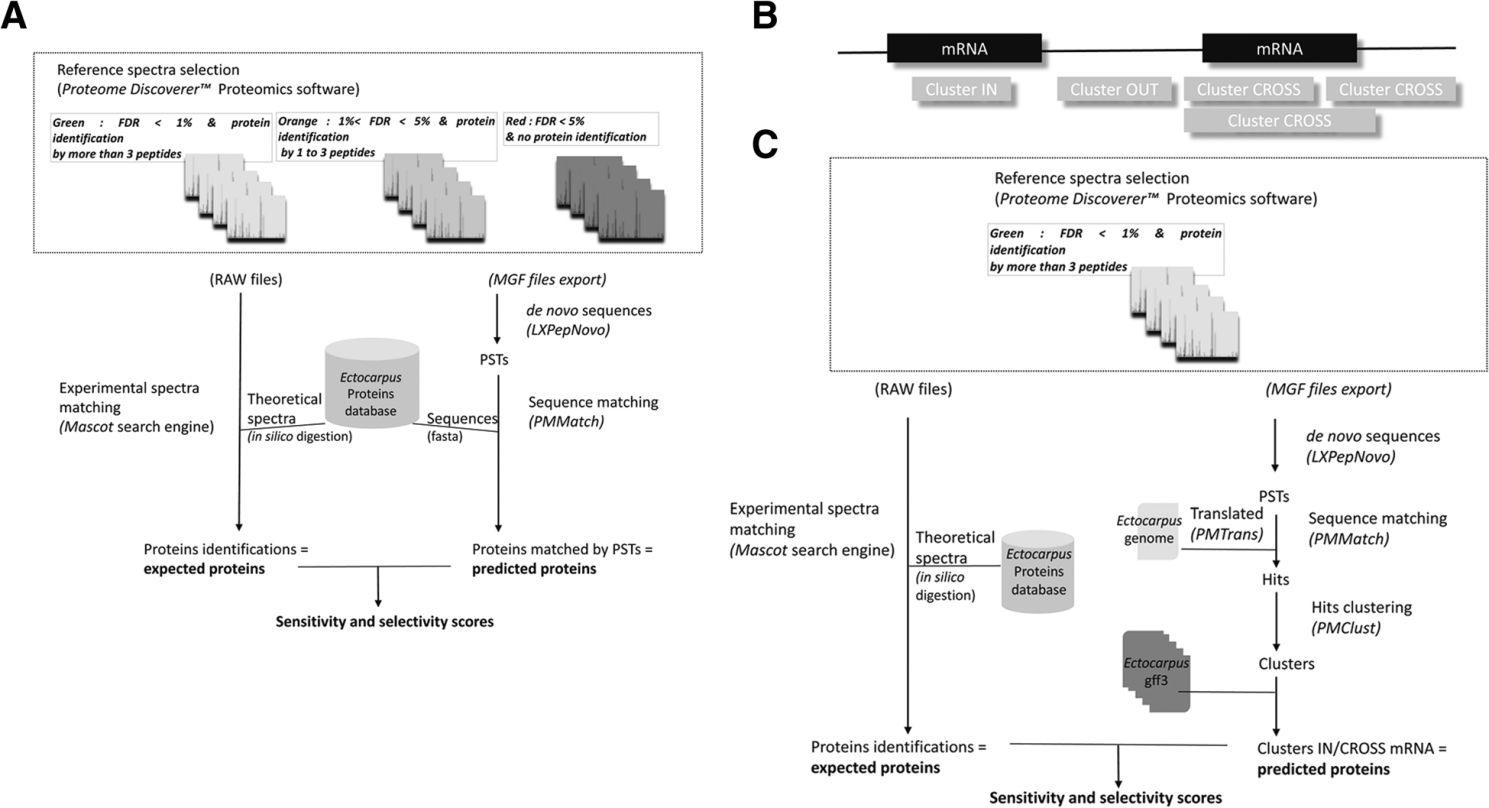
**a** Mapping step testing method. Predicted proteins matched by PSTs were compared to the expected proteins, identified by conventional database spectral identifications, using the same spectra and protein sequences database (ORCAE). **b** Definition of the cluster exon-mapping categories according to their mRNA locations (*Ectocarpus sp.* GFF3 files, ORCAE): *IN* when clusters were located inside an mRNA feature, *OUT* when clusters were located outside an mRNA feature, and *CROSS* when clusters were located across an mRNA feature. **c** Clustering step testing method. We only used the green dataset. The number of predicted proteins (matched by *IN* and *CROSS* clusters) was compared to that of expected proteins identified with *Proteome Discoverer™* using the *Ectocarpus sp.* database (ORCAE)

The second step consisted of successive genome translation, PST mapping, and hits clustering. We separately tested *PMMatch* and *PMClust* scripts, which were then grouped together into *LXPepMatch* (Fig. 2*)*. We first generated PSTs from the three previously defined reference datasets (see Additional file 2). For PST mapping, we adapted *PMMatch*, which is a module of PepLine [18] designed to locate PSTs on complete genome sequences, by adding an option to specify an absolute mass tolerance. It was set to 0.5 Da for our test case. We defined the PST length (i.e., optimal number of amino acids) and compared the results with those for which one or no amino acid modifications were allowed, by mapping PSTs of each reference spectra dataset against the *Ectocarpus sp.* gene model proteins (downloaded from ORCAE, Ectsi_prot, 2010) (Fig. 3a). Proteins matched by PSTs were compared to the expected proteins identified by conventional database spectral identification, using the same spectra and same protein sequence database (Ectsi_prot, 2010) with *Proteome Discoverer™* 1.2 software. A hit (the location of a PST on the genome sequence) was considered to be valid if it matched the expected protein. We considered proteins matched by at least two valid hits and corresponding to expected proteins to be true-positives (“*true_pos*”). We defined “*nref”* as the number of expected proteins and “*nfound”* as the number of proteins matched by *PMMatch*. Sensitivity was defined as the percent of expected proteins matched (*true_pos*/*nref)* and selectivity the percent of expected proteins among all proteins matched using our workflow (*true_pos*/*nfound).* We computed these metrics for each reference dataset by varying tag lengths from 3 to 5.

For hit clustering, we only used the green reference dataset to test the program *PMClust*, another module of PepLine. We optimized this step by selecting the maximal distance between two consecutive hits to be grouped in a cluster, taking into account the mean length of CDSs, exons, introns, genes, and intergenic regions of the *Ectocarpus sp.* genome. We defined the minimum number of hits (*MINHIT*) and minimum number of peptides (*MINPEP*) a cluster could contain to improve the results. Clusters were mapped to the 1591 annotated contigs (GFF3 files downloaded from ORCAE, Ectsi_gff3, 2011) and classified into three categories, according to their mRNA locations: *IN* when clusters were located inside an mRNA feature, *OUT* when clusters were located outside of an mRNA feature, and *CROSS* when clusters were located across an mRNA feature (Fig. 3b).

We performed the first test by analyzing their distribution into each category for various values of *MINHIT* and *MINPEP*, with the aim of obtaining the maximum number of *IN* clusters. In the second test, we compared proteins that matched *IN* and *CROSS* clusters to the expected proteins identified by conventional database spectral identification, using the same spectra and protein sequence database (Ectsi_prot, 2010) with *Proteome Discoverer™* 1.2 software (Fig. 3c). We considered proteins matched by an *IN* or *CROSS* cluster and corresponding to an expected protein to be true-positives (“*true_pos*”). All *IN* and *CROSS* clusters corresponded to found proteins (*“nfound”),* whereas all expected proteins corresponded to proteins identified by the database-driven approach (*“nref”)*. We calculated the sensitivity (*“true_pos”/“nref”)*, which is the percent of expected proteins matched by an *IN* or *CROSS* cluster among all expected proteins, and the selectivity, which is the percent of expected proteins among all proteins found by *IN* or *CROSS* clusters (Fig. 3c). We computed these metrics using the green reference dataset for various values of *MINHIT* and *MINPEP*.

#### Step 3: Annotation and visualization of cluster results

In the third step (Fig. 2), we developed a specific script, *LXQualify*, allowing the annotation of clusters with three labels. The first was “*UNANNOTATED”* or “*ANNOTATED”,* if no hits or at least one hit was included in a CDS of a protein-coding gene, respectively. The second was “*DUBIOUS*”, “*POSSIBLE*”, or “*SURE*”, to assign a confidence degree to each cluster according to the number of typical spectra (i.e.*,* specific to the cluster) and the number of hits. A cluster was “*DUBIOUS”* if it contained zero or one typical spectrum; “*POSSIBLE”* if it contained two or more typical spectra, but less than three different peptides, and “*SURE*” if it contained two or more typical spectra and three or more different peptides. The third was “*OK*” or “*CHECK*”, if all the hits were included in an annotated CDS or at least one hit did not match with the annotated CDS, respectively. Labeled clusters were listed in a tabular output file.

We developed a specific script, *LXClust2Gff*, to convert *PMClust* output to GFF files for visualization. Artemis (Sanger Institute, England) [27] was used as the genome sequence viewer to conveniently assess our results.

Some clusters of interest were validated by comparison with transcriptomics data and RT-PCR experiments. Transcriptomic data (ESTs and RNA-Seq coverage) were downloaded from the ORCAE public database of the *Ectocarpus sp.* genome. These data were previously published with research articles [32, 33]. For RT-PCR experiments, approximately 100 mg wet weight of frozen samples of *Ectocarpus sp..* Ec 32 were quickly ground in liquid nitrogen for RNA extraction. Total RNA was prepared as described previously [34]. RNA quantity and quality were verified using a NanoDrop ND-1000 spectrophotometer (NanoDrop products, Thermo Fisher Scientific) and by electrophoresis on agarose gels. cDNAs were produced from 1 μg total RNA using the ImProm-II™ Reverse Transcription System (Promega, Charbonnières-les-Bains, France). PCR experiments were carried out with a thermocycler machine using a standard GoTaq® DNA Polymerase Protocol (Promega). The annealing temperature of the specific primers for cluster validation were between 58 and 60 °C. PCR products were separated and purified on agarose gels. The DNA was subsequently sequenced using the BigDye® Terminator v3.1 Cycle Sequencing Kit and a 3130 Genetic Analyzer (Applied Biosystems, Foster City, California, USA).

## Results

We designed and built our proteogenomics tool using the brown alga *E. sp.* as our test case. The *Ectocarpus sp.* (formerly included in *E. sp.,* [35]) has become a model organism for brown algal biology because of its amenable features for morpho-genetic, life-cycle, and genetic studies. Publication of the *Ectocarpus sp.* genome in 2010 propelled brown algal research into the genomic era and several post-genomic tools have been subsequently developed using this species to explore diverse aspects of brown algal biology, including its life cycle, development, metabolic processes, and interactions with the environment [32, 36]. Resources for *Ectocarpus sp.* now include two genetic maps [37, 38], gene mapping techniques, microarrays [39, 40], transcriptomic data [41, 42], proteomic techniques [43, 44], and bioinformatics tools for the prediction of peptide addressing [45] and metabolic reconstruction [46].

### Workflow: settings and test results

We optimized our proteogenomics approach (Fig. 1a) using the three reference datasets. It required fine-tuning for the type and quality of the MS data and adaptation to the characteristics of the *Ectocarpus sp.* genome. The initial genome V1 annotation retrieved 16,256 protein-coding sequences, among which 6655 had no EST support and 5819 concerned specific brown algal genes encoding proteins with no known function [32]. Moreover, the high number of introns per gene (an average of seven), the extended 3’UTR regions, with an average length of 845 bp, and short intergenic regions often hampered accurate gene prediction.

### Step 1: From MS/MS spectra to PSTs

We selected the best from among three programs (i.e.*, Taggor*, *Peaks*, *PepNovo+*) to generate PSTs from MS/MS spectra. *Taggor* had difficulties distinguishing doubly charged ions from singly charged ions, leading to sequence errors. This tool also required a preliminary deconvolution step. We thus discarded it and focused on *Peaks* and *PepNovo+*. Ten high quality experimental spectra identified by *Proteome Discoverer™* 1.2 software (see Additional file 2) were manually selected for use as reference sequences (Table 1) to cross-reference PST sequences generated by each program we tested.

**Table 1:** Comparison of tag quality between *Peaks* and *PepNovo+*, using *Proteome Discoverer* ™ peptide identifications as the expected sequences

Peaks				Proteome discoverer (MASCOT identifications)			Pepnovo+		
ID	Tag sequence	Significant score >60%	Rank	Protein AC	Reference Sequence	e-value	ID	Tag sequence	Significant score >1,5
271	WVQAAGAGASR	23	0	Esi0085_0010	**SVVQAAGAGDAK**	5,65568E-06	Spectrum271_scans	**QAAGAGA**	6,346
271	WVQAAGAGWK	21.5	1						
271	WVQAAGAGTGR	18.5	2						
552	(CamC)VGVSEETTTRHR	31	0	Esi0091_0058	**cVGVSEETTTGVHR**	2,36909E-07	Spectrum552_scans	GVSEDD	4,237
552	QMGVSEETTTRHR	29	1						
552	V(CamC)GVSEETTTRHR	17	2						
577	QFAGDDAPR	43	0	Esi0203_0038	**AGFAGDDAPR**	1,69937E-06	Spectrum578_scans	**GDDAPR**	7,539
577	K(MetOxM)AGDDAPR	41	1						
577	**AGFAGDDAPR**	11.5	2						
642	KAENPMSKR	100	0	Esi0349_0012	**KAEDIDTIR**	1,01272E-05	Spectrum643_scans	DLDTLR	4,727
642	KAQDPMSKR	0.1	1						
642	QAKDPMSKR	0.02	2						
1118	DGLVYGK(MetOxM)NEPPGAR	38	0	Esi0327_0021	**ATLVYGQmNEPPGAR**	2,42457E-06	Spectrum1119_scans	**EPPGAR**	1,396
1118	DGLVYGQFNEPPGAR	37	1						
1118	DGLVYGQ(MetOxM)NEPPGVK	8	2						
2462	**DESAAVFAWK**	87,5	0	Esi0091_0058	**DESAAVFAWK**	1,64304E-05	Spectrum2463_scans	**SAAVFA**	4,78
2462	DESAAVFAGTR	8	1						
2462	LMSAAV(MetOxM)AGEK	1.5	2						
2479	MDDLTNNALARK	58	0	Esi0888_0002	**mVDLTPMAIAAGR**	4,70366E-07	Spectrum2480_scans	**ALAAGR**	1,943
2479	(MetOxM)VDLTNNALAAGR	39.5	1						
2479	MDDLTNGGALARK	1	2						
2558	ED(MetOxM)ETE(CamC)AVNYDNLYQVMK	48	0	Esi0349_0012	**TccEAEcAVNYDNLYQAMR**	1,01272E-05	Spectrum2559_scans	**VNYDNL**	2,289
2558	DE(MetOxM)ETE(CamC)AVNYDNLYQVMK	8.5	1						
2558	ED(MetOxM)ETE(CamC)AVNYDNLYQMVK	8	2						
2610	TFQAGEVASALLGR	32	0	Esi0327_0021	**FTQAGAEVSALLGR**	5,99619E-08	Spectrum2610_scans	**SALLGR**	4,356
2610	**FTQAGEVASALLGR**	25	1						
2610	TFKAGAGNGSALLGR	10	2						
3191	**VALTGLTLAEYFR**	53	0	Esi0327_0021	**VALTGLTIAEYFR**		Spectrum3196_scans	**LAEYFR**	4,471
3191	GLLTGLTLAEYFR	25.5	1						
3191	AVLTGLTLAEYFR	11	2						

The *Peaks* and *Pepnovo* + results are shown in the first and the last columns, with the *Proteome Discoverer* ™ identifications in between as reference sequences. We ran *Peaks*, *PepNovo*+, and *Proteome Discoverer* ™ on the top 10 high-quality selected spectra. Each spectrum corresponds to one row in the table. Sequences shown in bold are the correct sequences, thus four for Peaks and eight for PepNovo+

*Peaks* generated PSTs of variable, generally long sequence-tag length (at least six amino acids) that could potentially lead to errors. Indeed, *Peaks* generated only four correct sequences (Table 1). The errors generated by *Peaks* are also explained by the mass tolerance accuracy parameter. For example, the amino-acid mass of “DD” is 230.05 Da and that of “ET” is 230.09 Da. The reference sequence tag to generate was “GVSEET”, whereas *Peaks* wrongly proposed “GVSEDD”. *PepNovo+*, raised two concerns. First, it did not take into account the H_2_O molecule plus the single charge acquisitions during the fragmentation process, resulting in errors in the mass of Mn NTer. Second, it did not take the peptide charge into consideration. Nevertheless, after correcting for these problems, *PepNovo +* appeared to be the best choice. Indeed, it returned 8 of the 10 reference sequences (Table 1). *PepNovo +* was set to two allowed amino-acid modifications, cysteine carbamidomethylation and methionine oxidation (C + 57 and M + 16, respectively). The maximal tag number generated per spectra was set to 10.

### Step 2. From PSTs to hit clusters

**Fig. 4:**
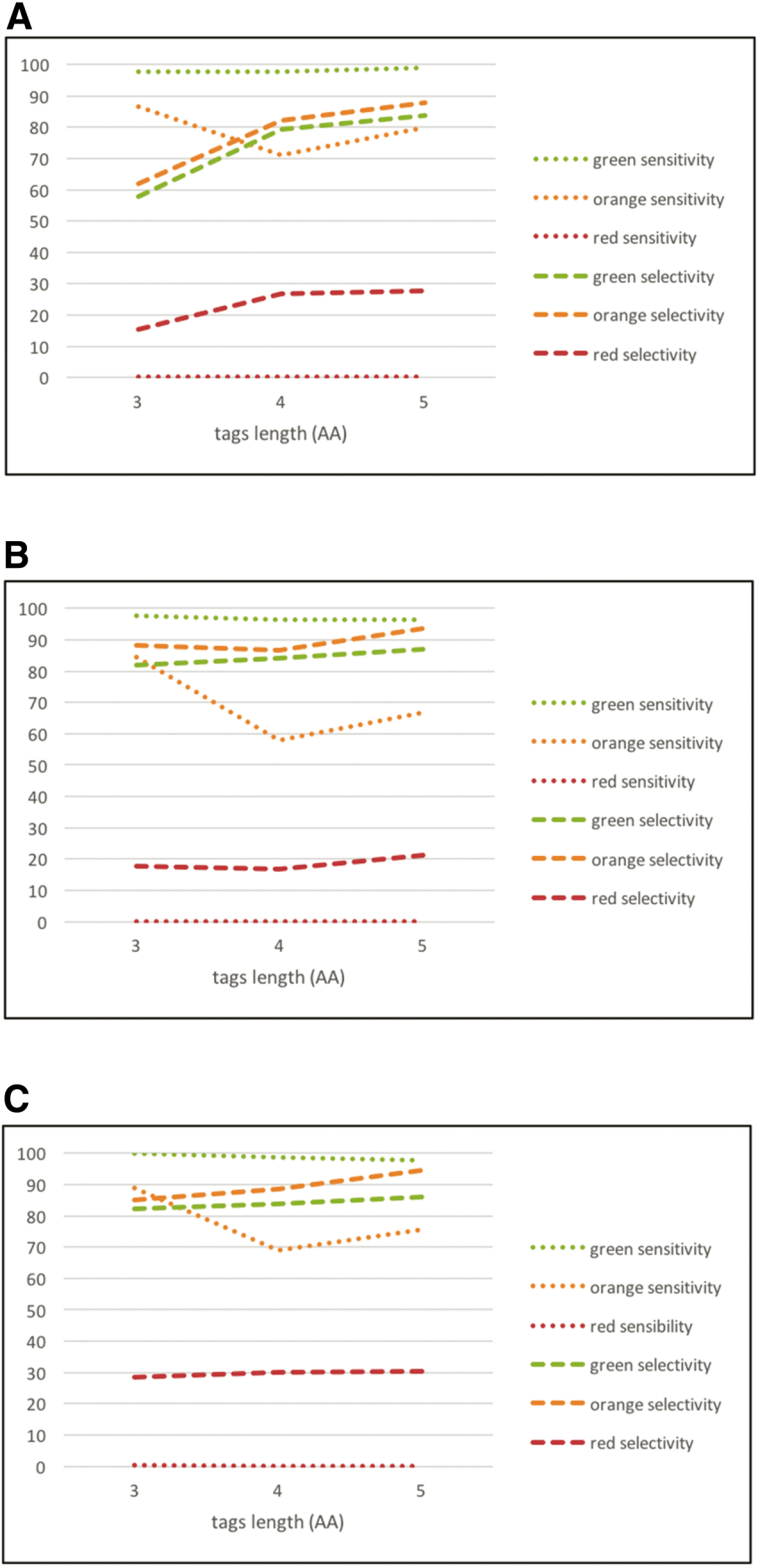
Sensitivity and selectivity were calculated for each dataset. Sensitivity measures the proportion of positives that are correctly identified by clusters and selectivity is the proportion of positives that are correctly identified among all proteins matched by clusters. **a** Selectivity and sensitivity for each reference dataset with *MINHIT* = 1. **b** Selectivity and sensitivity for each reference dataset with *MINHIT* = 2. **c** Selectivity and sensitivity for each reference dataset with *MINHIT* = 2 and one amino acid modification allowed

PST mapping was performed using the *PMMatch* program. The results of three PST files generated by *PepNovo +* from the *green*, *orange,* and *red* reference spectra datasets (see Additional file 2) were used as input. We then compared protein encoding genes matched by PSTs to the expected proteins identified by *Proteome Discoverer™* from the same spectra dataset, using the same *Ectocarpus sp.* protein database (ORCAE, Ectsi_prot, 2010) as that used by *PMMatch* (Fig. 3a). Sensitivity and selectivity were calculated for each dataset. Sensitivity measures the proportion of positive IDs that were correctly identified among all expected proteins and selectivity the proportion of positive IDs that were correctly identified among all matched proteins. The results of each reference dataset are reported in Fig. 4 for three different sequence tag lengths (i.e., 3, 4, or 5 amino acids) for a minimum of one or two hits per protein (*MINHIT*) with one or no amino acid modifications allowed.

We observed poorer selectivity and slightly better sensitivity for the high and medium quality spectra with *MINHIT* = 1 (Fig. 4a) than with *MINHIT* = 2 (Fig. 4b). *MINHIT* = 2 resulted in poorer sensitivity for noisy spectra. Thus, optimal parameters depend on spectra quality. Selectivity increased with PST length, regardless of the quality of the spectra, with a slight unfavorable influence on sensitivity, particularly for spectra of medium and poor quality. Thresholds of *MINHIT* > 1 and a tag sequence length = 5 amino acids allowed us to focus on spectra of relatively good quality. We thus reduced the number of results to be validated from our preliminary study. Sensitivity and selectivity were higher when *MINHIT* = 2 and one amino acid modification was allowed, regardless of the quality of the spectra (Fig. 4c). We thus set allowed amino-acid modifications to one for PST mapping.

**Fig. 5:**
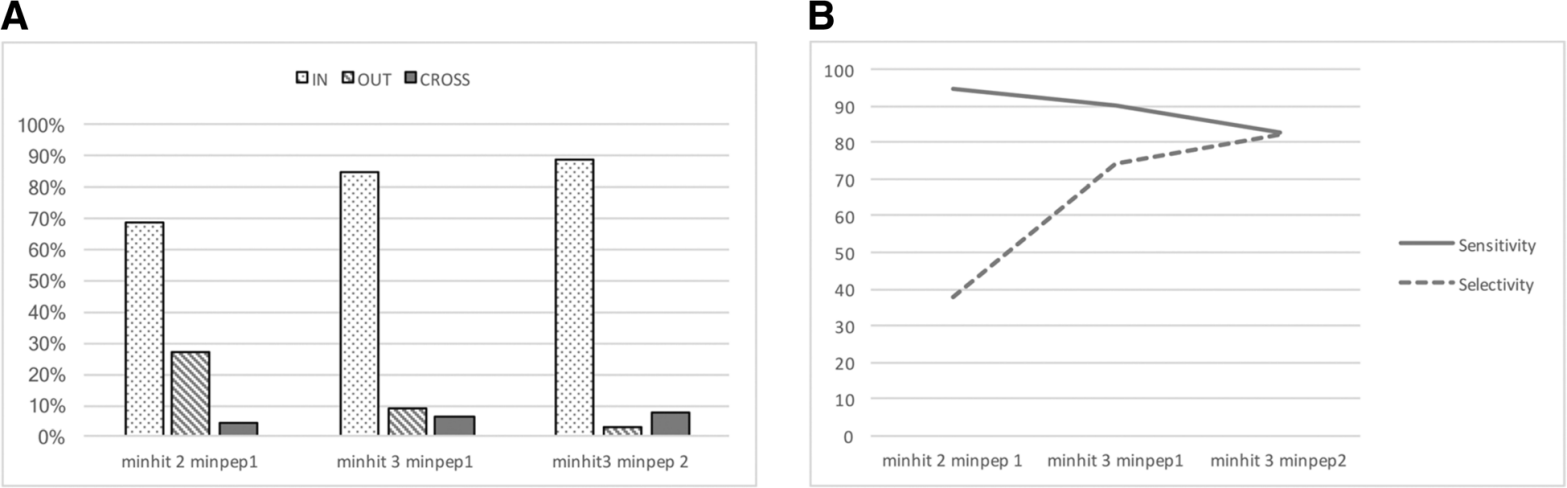
**a** Percentage of clusters qualified as *IN*, *OUT*, or *CROSS* with respect to predicted gene locations from *Ectocarpus sp.* GFF3 files (ORCAE). **b** The number of proteins (matched by *IN* and *CROSS* clusters) relative to the number of expected proteins (identified with *Proteome Discoverer™*) allowed the calculation of sensitivity and selectivity. Sensitivity measures the proportion of positives that are correctly identified by IN or CROSS clusters. Selectivity corresponds to the proportion of positives that are correctly identified among all proteins matched by IN or CROSS clusters

We performed further tests on the cluster results to set the optimal minimum hits per protein parameter (*MINHIT*). We compared proteins matched by clusters to the expected proteins identified by conventional database spectral identification using *Proteome Discoverer™* 1.2 software and the same spectra and protein database as above (ORCAE, Ectsi_prot, 2010) (Fig. 3c). We generated PSTs from only the *green* reference dataset, running *PMMatch* on the *Ectocarpus sp.* genome sequence (ORCAE, Ectsi_genome_V2_cleaned.tfa) translated by *PMTrans*. We clustered hits with the aim of uncovering regions potentially associated with genes or, at least, exons. The maximal distance between two consecutive hits in a cluster was correlated with *E. sp.* genome features to improve cluster results. The statistical distribution was established for each, starting from the *E. sp.* GFF3 files (ORCAE, Ectsi_gff3, 2011; Ectsi_genome_V2_cleaned.tfa): CDS (median of 137 nt), exons (median of 143 nt), introns (median of 531 nt), gene (median of 4772 nt), and intergenic regions (median of 2529 nt). We observed relatively short CDS and introns and short intergenic regions, with a median that was only four times larger than that of introns. Thus, there was a risk of confusing introns and intergenic regions. We thus fixed the maximal distance between hits to 5000 nt, thus minimizing the risk to merge two proteins while covering 99.6% of introns. Each cluster was annotated to fall into one of three categories, “*IN*”, “*OUT*”, or “*CROSS*”, according to *Ectocarpus sp.* mRNA locations (Fig. 3b), to further set the minimal number of hits (*MINHIT*), and validated peptides (*MINPEP*) required to form a cluster. First, we assessed the proportion of clusters falling into each category and the best result, i.e.*,* 90% of the clusters obtained were “IN” with a minimum of three hits and two peptides (Fig. 5a). Second, we compared the number of proteins predicted by the “*IN*” and “*CROSS”* clusters to the expected number of proteins (i.e., identified with *Proteome Discoverer™* using the same reference spectra) for different values of *MINHIT* and *MINPEP* (Fig. 5b). We obtained satisfactory sensitivity and selectivity scores of 83 and 82%, respectively, with a minimum of three hits and two peptides. Last, we ran *PMClust* with the following parameters: *MINHIT* at three hits, *MINPEP* at two peptides, and the maximal distance between two hits to form a cluster of 5000 nucleotides.

### Final workflow: validation on all samples of *Ectocarpus sp.*

We produced a PST file from each biological sample using *LX_RunPepNovo*. PSTs were mapped on the six-frame translations of the *Ectocarpus sp.* genome (ORCAE, Ectsi_genome_V2_cleaned.tfa) using the *LXPepMatch* program with the optimized parameters described above, thus generating 20-hit lists that were pooled to obtain only one file per sample. We used various strains to isolate biological samples of *Ectocarpus sp*.. We thus needed to avoid mistakes linked to small genetic differences due to polymorphisms. Before clustering, we merged hits files of each strain: Ec32 (soluble, membrane, and cell wall fractions), Ec594 (gametophyte and nuclei fractions), and Ec410 (gamete fraction). We generated clusters from each hits file, i.e., Ec32, Ec594, and Ec410, with the optimized parameters described above, using GFF3 files (ORCAE, Ectsi_gff3_Jun2013).

**Fig. 6:**
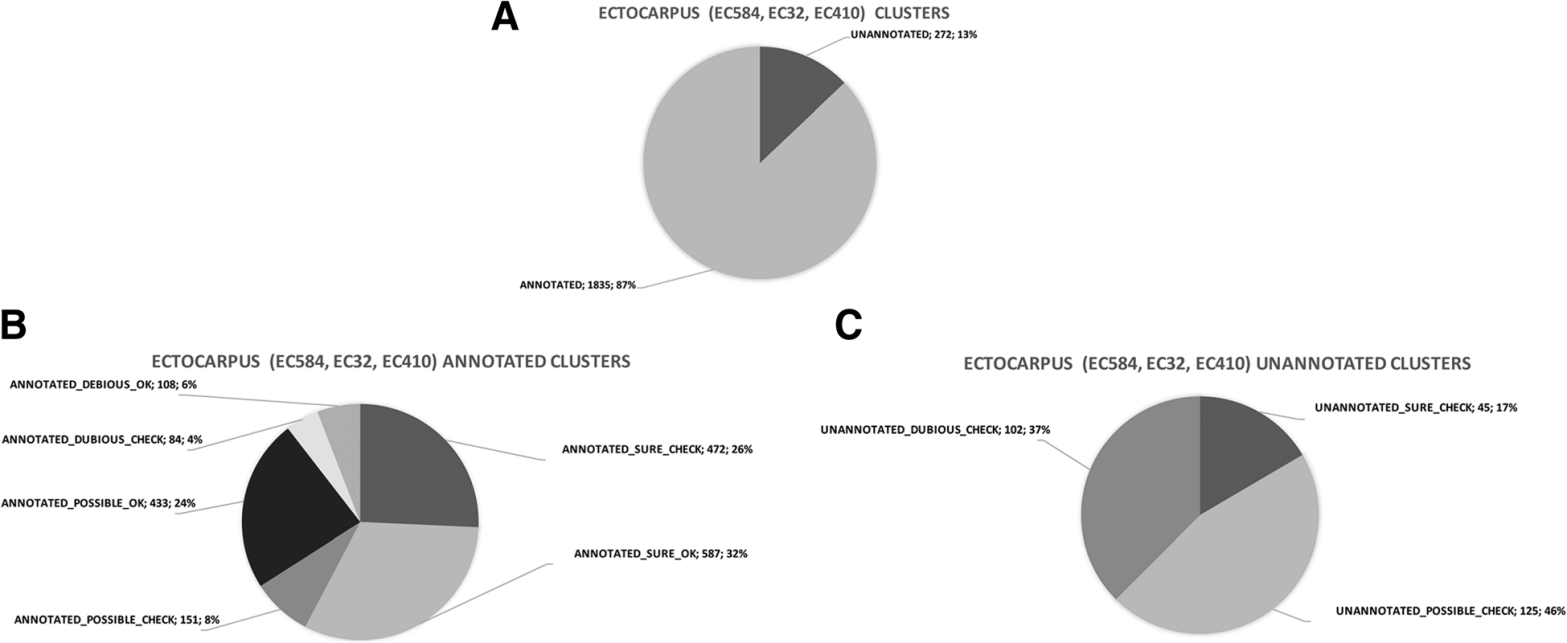
**a** Distribution of all *ANNOTATED/UNANNOTATED Ectocarpus sp.* clusters. **b** Distribution of *ANNOTATED* clusters according to category. **c** Distribution of *UNANNOTATED* clusters according to the category

The resulting list contained 2107 unique clusters (see Additional file 3), combining all strains, that included 272 unannotated and 1832 annotated clusters. We further analyzed a subset of these clusters. We annotated clusters to fall into one of three grades to assign a degree of confidence to each, based on the number of typical spectra (i.e.*,* specific to the cluster) and the number of hits, as described above. Thirteen percent of clusters were unannotated and 87% annotated (Fig. 6a). We focused on *SURE* or *POSSIBLE* and *OK* or *CHECK* clusters (see implementation step 3).

We validated our workflow by focusing on distinct case-results. i) We studied clusters annotated as *SURE* and *CHECK* to correct mispredicted genes or correct the ATG start codon. We found 472 clusters annotated as *SURE* and *CHECK* (Fig. 6b). Among these, two were retained for further experimental validation. ii) We only focused on the 45 unannotated clusters labeled *SURE* (Fig. 6c) to find new CDSs. Among these, two were further analyzed.

### Correction of mispredicted genes or ATG start codons

**Fig. 7:**
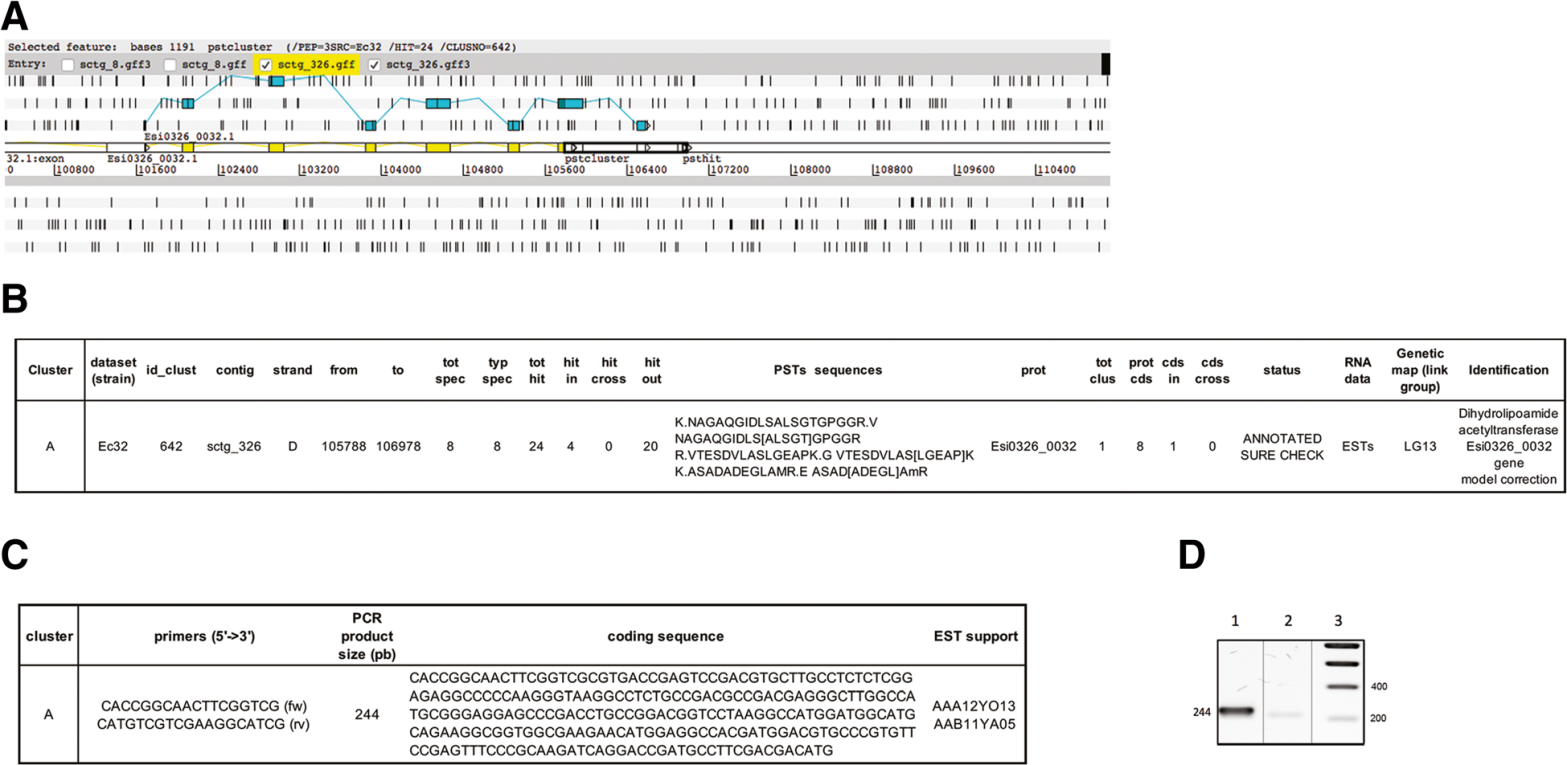
**a** Artemis view of cluster “A” for validation. *Ectocarpus sp.* genes close to these clusters are represented by linear exons colored in yellow. Another representation of the same exons is shown in light blue along the six reading frames (+ 1, + 2, + 3 above and − 1, − 2, − 3 below). The cluster is indicated by black bold rectangles around the small rectangles of the PSTs. **b** Data for cluster “A”. **c** Experimental validation conditions. **d** RT-PCR validation: agarose gel electrophoresis of RT-PCR products (lane 1), RNA-PCR negative controls (lane 2), and DNA size marker (lane 3) for PST cluster “A”

The first cluster, named “cluster A”, was present on sctg_326 between 105,788 and 106,978 (Fig. 7a). It was “*ANNOTATED_SURE_CHECK”* and only identified in Ec32 samples. This cluster was positioned downstream of the predicted gene model Esi0326_0032, annotated as a dihydrolipoamide acetyltransferase. A blastX search (on the ORCAE website, http://bioinformatics.psb.ugent.be/blast/moderated/?project=orcae_Ectsi, Ectsi_genome11x) against all portions of the Esi0326_0032 gene followed by the DNA sequence of cluster “A” identified a full-length protein for the dihydrolipoamide acetyltransferase component of the pyruvate/2-oxoglutarate dehydrogenase complex. This was also corroborated by two ESTs: AAA12YO13, AAB11YA05 (ORCAE, Ectsi_ESTs_cleaned) matching this region (Fig. 7b). Our results showed that the prediction of the last exon of the Esi0326_0032 gene model appeared to be false. Consequently, we selected this cluster as a candidate for correction of a mispredicted gene. We designed primer pairs to amplify portions based on the sequences of the PSTs for validation (Fig. 7c), as public databases suggested that the “A” cluster was expressed in vivo by *E. sp.*. PCR products of the expected size of 244 bp were obtained (Fig. 7d) and sequencing confirmed the presence of the expected nucleotide sequences (data not shown).

**Fig. 8:**
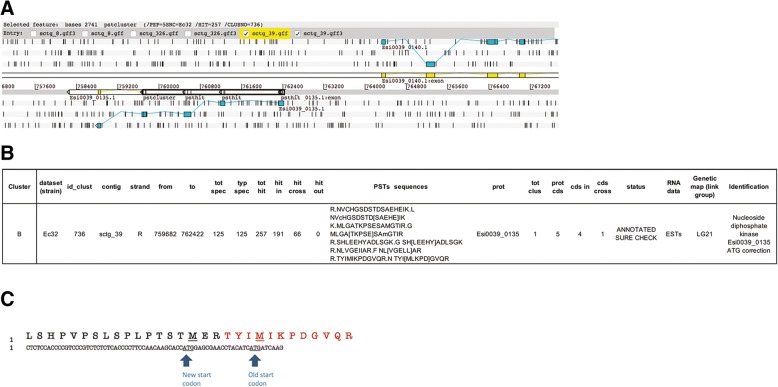
**a** Artemis view of cluster “B” for validation. *Ectocarpus sp.* genes close to these clusters are represented by linear exons colored in yellow. Another representation of the same exons is shown in light blue along the six reading frames (+ 1, + 2, + 3 above and − 1, − 2, − 3 below). The cluster is indicated by black bold rectangles around the small rectangles of the PSTs. **b** Data for cluster “B”. **c** Translation view of the 5’ cDNA sequence corresponding to the region defined by the cluster

The second cluster, named “cluster B” was present on sctg_39 between 759,682 and 762,422 (Fig. 8a). It was “*ANNOTATED_SURE_CHECK”* and only identified in Ec32 samples*.* Most (191 “hit in”) of the total hits (257 “tot hit”) were present in the predicted sequence of an *Ectocarpus sp.* gene corresponding to Esi0039_0135 (Fig. 8b). These 191 hits corresponded to five peptides located in the region of the predicted Esi0039_0135 protein-coding gene (“cds in”). Another peptide, “TYIMIKPDGVQR”, partially covered the Esi0039_0135 sequence (“cds cross”). The predicted start codon of the Esi0039_0135 protein-coded gene is included in this peptide sequence and further analysis of the ESTs showed that the true start codon of this gene may likely be upstream of the predicted one (Fig. 8c). Indeed, these two potential start codons are very close (separated by five amino acids) emphasizing the benefit of a proteomic approach for true start codon assignment. The identification of the PST sequence TYIMIKPDGVQR in the MS/MS data led us to propose a new position for the ATG start codon of the Esi0039_0135 gene. Nevertheless, we cannot exclude that the two ATG codons may be alternatively used in vivo to produce different translation products of this nucleoside diphosphate kinase.

### New CDS discovery

**Fig. 9:**
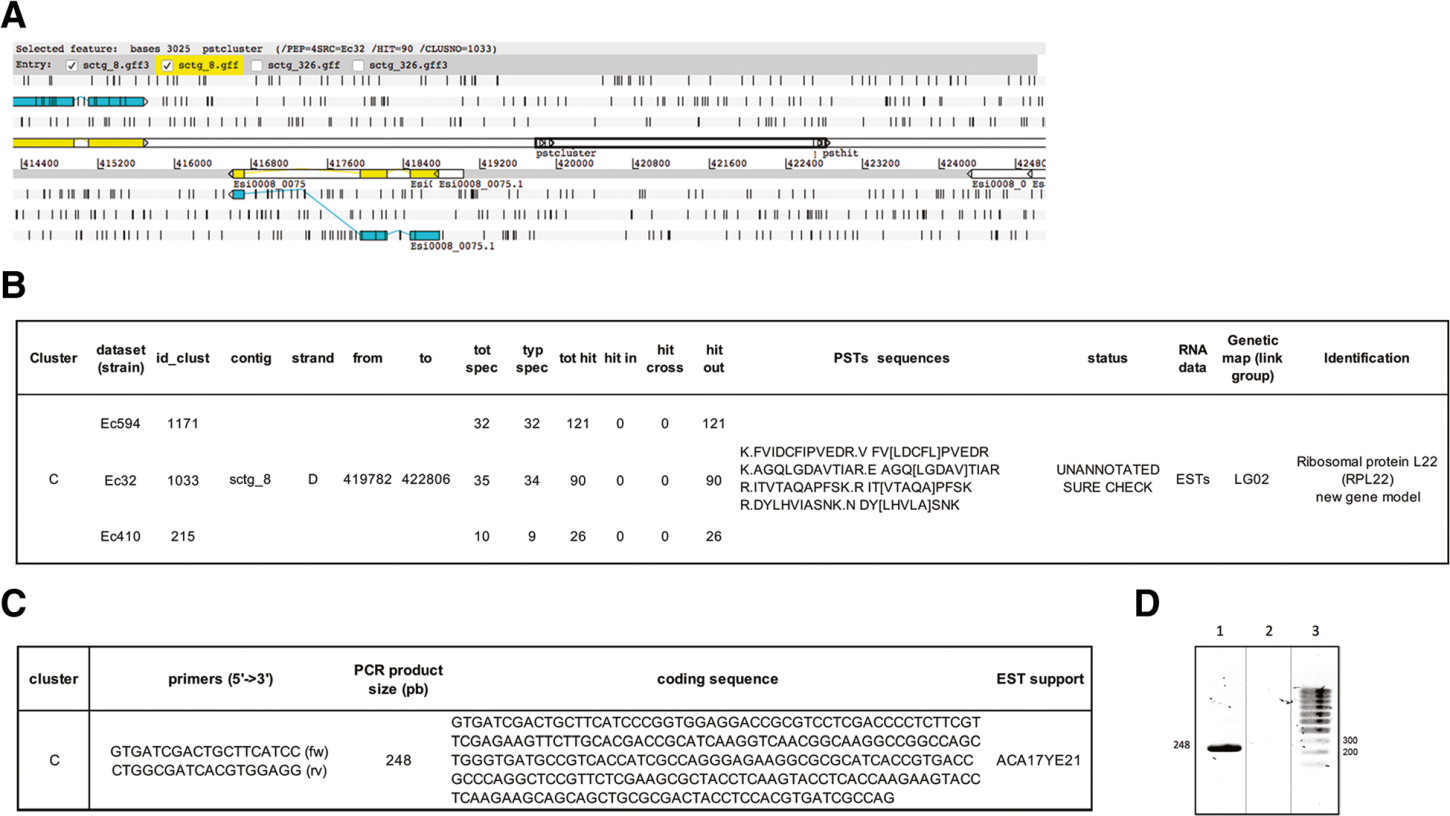
**a** Artemis view of cluster “C” for validation. *Ectocarpus sp.* genes close to these clusters are represented by linear exons colored in yellow. Another representation of the same exons is shown in light blue along the six reading frames (+ 1, + 2, + 3 above and − 1, − 2, − 3 below). The cluster is indicated by black bold rectangles around the small rectangles of the PSTs. **b** Data for cluster “C”. **c** Experimental validation conditions. **d** RT-PCR validation: agarose gel electrophoresis of RT-PCR products (lane 1), RNA-PCR negative controls (lane 2), and DNA size marker (lane 3) for PST cluster “C”

“Cluster C”, was mapped against sctg_8 from position 419,782 to position 422,806 (Fig. 9a) with four peptides (Fig. 9b). It was “*UNANNOTATED_SURE_CHECK*” and observed in the three datasets. An MS-Blast search [47] (http://genetics.bwh.harvard.edu/msblast/), using PST sequences included in this cluster, revealed similarity with ribosomal protein L22 (RPL22) from other species. In addition, cross-analysis with *Ectocarpus sp.* transcriptomic data available from the ORCAE website (http://bioinformatics.psb.ugent.be/blast/moderated/?project=orcae_Ectsi) showed that a complete cDNA sequence (ACA17YE21) was present in cluster “C”. This sequence appears to be a good candidate for a new protein coding gene as no coding gene has yet been predicted in this region (Fig. 9a). We designed primer pairs to amplify portions based on the PST sequences (Fig. 9c) for validation, as transcriptomic data in public databases suggested that the “C” cluster is likely to be expressed in vivo in *E. sp.*. PCR products of the expected size of 248 bp were obtained (Fig. 9d) and sequencing confirmed the presence of the expected nucleotide sequences (data not shown).

**Fig. 10:**
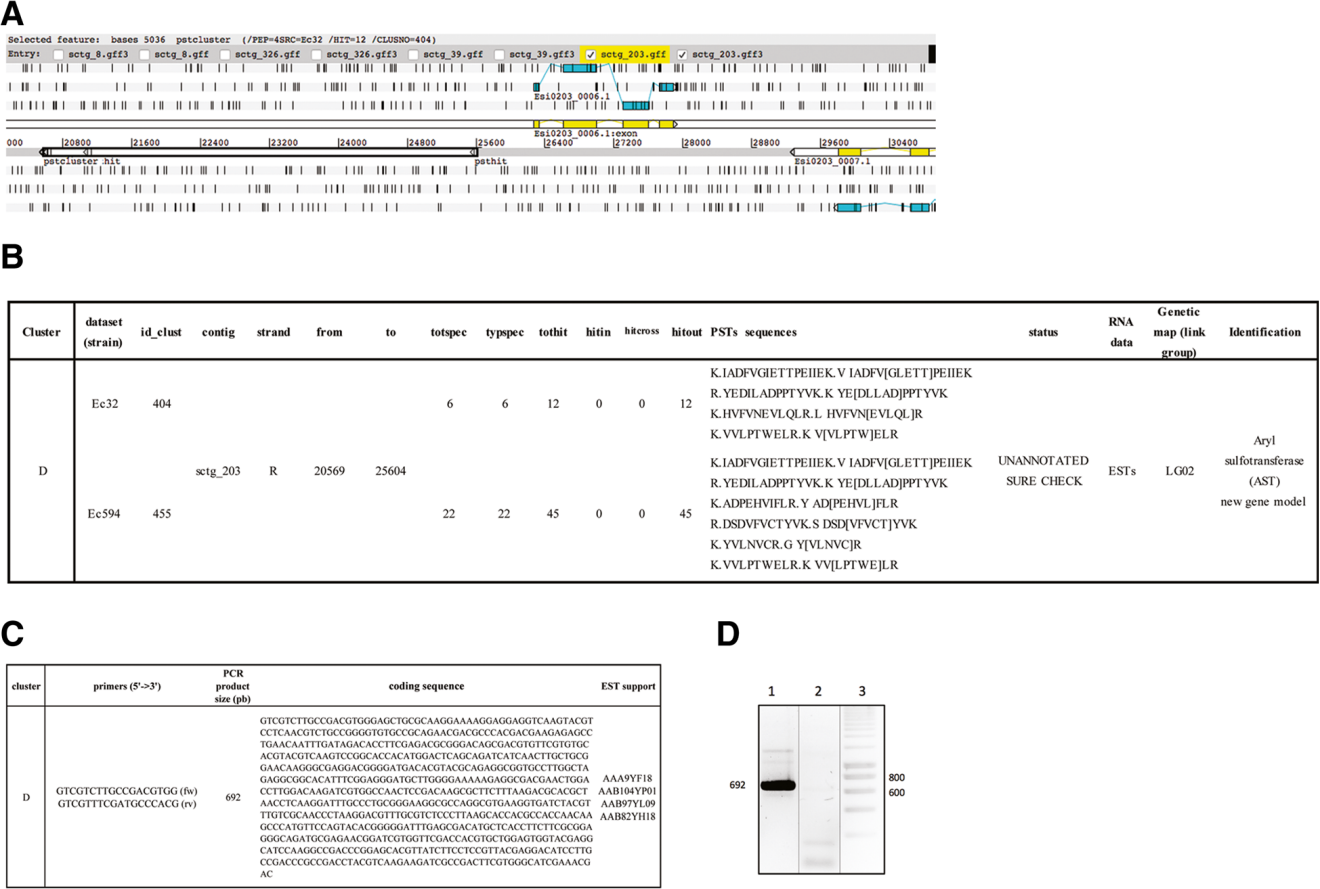
**a** ARTEMIS view of cluster “D” for validation. *Ectocarpus sp.* genes close to these clusters are represented by linear exons colored in yellow. Another representation of the same exons is shown in light blue along the six reading frames (+ 1, + 2, + 3 above and − 1, − 2, − 3 below). The cluster is indicated by black bold rectangles around the small rectangles of the PSTs. **b** Cluster “D” data. **c** Experimental validation conditions. **d** RT-PCR and EST validation: agarose gel electrophoresis of RT-PCR products (lane 1), RNA-PCR negative controls (lane 2), and DNA size marker (lane 3) for PST cluster “D”

The last cluster, named “cluster D”, was present on sctg_203 between 20,569 and 25,604 (Fig. 10a). It was observed in two datasets (Ec32 and Ec594) and is present on LG02 of the genetic map [38] (Fig. 10b). We also selected it for validation by RT-PCR analysis (Fig. 10d). We designed PCR primers based on the coding sequences of the most distant PSTs identified for this cluster (VVLPTWELR and IADFVGIETTPEIIEK). In addition, four ESTs were found to cover the entire length of the cluster. Amplification of *Ectocarpus sp.* cDNAs led to an expected product of 692 bp absent from the RNA amplification negative control, confirming the translation of this new gene product (Fig. 10d).

### Peptimapper workflow distribution

Our proteogenomics workflow, Peptimapper, is composed of four scripts from the *Ectoline* github project: *LXRunPepNovo*, *LXPepMatch*, *LXQualify*, and *LXClust2Gff* (Fig. 2; see command line arguments and output file descriptions into Additional file 4). *PMMatch*, used by *LXPepMatch*, was adapted from the Pepline suite (version 2.0.1) to fit our workflow. *LXRunPepNovo* is a new version of *PepNovo* + (version 3.1 beta), adapted for our data. This version contains sources and pre-compiled binaries for Linux and MacOS platforms. *Ectoline* project is distributed under the GPL or CECILL license. The text of both licenses is attached (and should remain attached) to this distribution and is available at https://github.com/laeticlo/Ectoline.

We built a Docker image, called peptimapper (see dockerfile into Additional file 5), to allow easier distribution and interoperability of *Ectoline* scripts. Anyone can retrieve this image from a public repository and run it as a package without any specific configuration or installation requirements. This package contains all workflow components (Fig. 2), along with dependencies and running environment. We use two different cloud-based public registry services for storing and distributing our Docker image: the Docker Hub (https://hub.docker.com/r/dockerprotim/peptimapper/) and BioShaDock [48], a public curated and bioinformatics-focused repository (https://docker-ui.genouest.org/app/#/container/dockerprotim/peptimapper). In the context of this project, every computational tool for each step of the overall workflow was integrated and deployed on our own Galaxy server https://galaxy.protim.eu, using this Docker image (see Additional file 5). Our workflow is therefore functionally reproducible with Galaxy [49, 50]. It ran on a virtual machine with 8 CPU and 70 Go RAM.

## Discussion

Most sequence-centric proteogenomics available pipelines are based on the generation of customized protein databases from genome, exome, or RNA sequencing to, e.g. reannotate genes, predict splice isoforms or discover novel proteins, using classical database-driven methods [7–9]. These methods are based on a direct comparison between experimental MS/MS spectra and theoretical MS/MS spectra generated from in silico digestion of these customized protein databases. A major advantage of such approaches is the specificity of the databases, including variations such as single amino acid variants (SAAVs) and alternative splice junctions. However, one of their weaknesses is the size of these databases, larger than those used in conventional proteomic searches and containing only known proteins. Consequently, it requires iterative search strategies and a specific FDR calculation to be sensitive enough to avoid false positive identifications [7]. Peptimapper overcomes this statistical drawback by first partially interpreting MS experimental spectra before mapping them onto the translated genome. Other similar pipelines currently exist that map MS-based proteomics data onto genomic coordinates as the Proteogenomic Mapping Tool [51], proteoAnnotator [52], PGMiner [53], Protk (https://github.com/iracooke/protk), IggyPep [54], or PepLine [18] (Table 2). However, for most of these pipelines, peptides are derived from database-driven methods, except for IggyPep and PepLine that also use de novo Peptide Sequence Tags (PSTs) obtained from partial interpretation of mass spectrometry data. Unfortunately, PepLine and IggyPep are neither maintained nor available anymore.

**Table 2:** Eukaryotic proteogenomics pipelines and Galaxy workflows

Name	Pipeline Interface	Database-driven for peptide identifi-cation	de novo peptide inter-pretation	User-friendly for biologists	Results curation	Results visuali-zation	Description	Revelance
Peptimapper (released in 2018)	Command line, Docker image, Galaxy tools	–	√	√	√	√	Peptide Sequence Tags (PSTs) obtained from partial interpretation of ion trap mass spectra are mapped onto the six-frame translation of genomic sequences giving hits. Hits are then clustered to detect potential coding regions. Clusters are evaluated and further compared to existing gene predictions. Clusters are available as GFF file to be uploaded into a genome viewer. https://galaxy.protim.eu https://hub.docker.com/r/dockerprotim/peptimapper/ or https://docker-ui.genouest.org/app/#/container/dockerprotim/peptimapper https://github.com/laeticlo/Ectoline	Improves genome annotation
IPAW (2018) [61]	Command line	√	–	–	√	–	This is an Integrated Proteomics Analysis Workflow: i) Peptide spectra are searched in two different databases in parallel: VarDB filtered by class-specific FDR for SAAV peptides and 6FT of the human genome filtered by peptides pI. ii) SAAV candidates are curated by SpectrumAI and potential novel proteins are blasted onto public databases. ii) Curated results are validated by different controls. https://github.com/yafeng/proteogenomics_python	Identification of Pseudogenes, lncRNAs, nsSNPs and somatic mutations
JUMPg (2016) [62]	Command line	√	–	–	√	√	This pipeline includes multiple customized databases construction, tag-based database search, peptide-spectrum match filtering, ans data visualization. https://github.com/gatechatl/JUMPg/	Improves genome annotation
PGMiner (2016) [63]	Command line	√	–	–	√	√	This workflow allows acquisition of mass spectrometric data, peptide identification against preprocessed sequence databases, assignment of statistical confidence to identified peptides, and mapping confident peptides to gene models. https://github.com/olalonde/pgtools	Improves genome annotation
PROTEO-FORMER (2015) [64]	Command line, Virtual machine, Galaxy tools	√	–	√	√	√	RIBO-seq NGS data are processed to delineates proteoforms. RIBO-seq-derived sequences are then translated and mapped to a public database, creating a custom search database for peptides to MS/MS matching.	Identification of novel translation products
PGTools (2015) [65]	Command line	√	–	–	√	√	The software is divided into 2 phases: Phase 1 contains 8 modules to analyse MS/MS data using known proteins databases. Phase 2 contains 5 modules and 7 customized databases that allow MS/MS data to be analysed against the genome. That software includes applications, libraries, customized databases and visualization tools.	Improves genome annotation
NextSearch (2015) [66]	Command line	–	–	–	√	√	Nucleotide EXon-graph Transcriptome Search identifies peptides by directly searching the nucleotide exon graph against tandem mass spectra. NextSearch outputs which are the proteome-genome/transcriptome mapping that can be visualized using public tools.	Improves genome annotation
ProteoAnnotator (2014) [52]	Command line, Stand alone application	√	–	√	√	√	MS spectrum are queried by one or several proteomics databases search engines (MASCOT, OMSSA, X!Tandem or MSGF+) and results are converted into GFF adding genome coordinates and statistical confidence values. It exports mzIdentML files. http://www.proteoannotator.org	Improves genome annotation
Peppy (2013) [67]	Command line, Stand alone application	√	–	N/A	√	–	This workflow generates a peptide database from a genome, tracks peptide loci, matches peptides to MS/MS spectra and assigns FDR confidence values to those matches. http://geneffects.com/peppy	Improves genome annotation
Protk (released in 2012)	Command line, Galaxy tools	√	–	√	–	√	It is a suite of tools for proteomics providing the following analysis tasks: (i) MS/MS data search with X!Tandem, Mascot, OMSSA and MS-GF+; (ii) peptide and protein inference with Peptide Prophet, iProphet and Protein Prophet; (iii) conversion of pepXML or protXML to tabular format, and (iv) mapping of peptides to genomic coordinates https://github.com/iracooke/protk	Improves genome annotation
IggyPep (2010) [54]	Web interface	√	√	N/A	–	–	The pipeline is based on a database system with advanced indexing and querying strategy, which holds the translated genome in all six reading frames. It can be queried with de novo sequences or partial peptide sequence tags (PSTs). It determines the ORF amino acid comprising these tags and compiles a fasta-formated sequence file for a database-driven search. www.iggypep.org (No more accessible)	Improves genome annotation
PepLine (2008) [18]	Command line	–	√	N/A	√	–	Peptide Sequence Tags (PSTs) obtained from partial interpretation of QTOF mass spectra are mapped onto the six-frame translation of genomic sequences giving hits. Hits are then clustered to detect potential coding regions. www.grenoble.prabi.fr/protehome/software/pepline (no more accessible)	Improves genome annotation
Workflows for Proteomics Informed by Transcriptomics (2015) [57]	Galaxy tools	√	–	√	√	√	Galaxy Integrated Omics (GIO) provides workflows for 4 common use cases: i) a standard search against a reference proteome; ii) PIT protein identification without a reference genome; iii) PIT protein identification using a genome guide; iiii) and PIT genome annotation. http://gio.sbcs.qmul.ac.uk	Improves genome annotation
Workflows for proteogenomics studies using Galaxy-P (2014–2018) [55, 56, 58, 59]	Galaxy tools	√	–	√	√	√	These modular workflows incorporating both established and customized software tools that improve depth and quality of proteogenomic results. http://galaxyp.org	Improves genome annotation

Available Eukaryotic Proteogenomics pipelines are listed in https://omictools.com/proteogenomics-category. We only selected software types “pipeline/workflow” or “Toolkit/Suite” for comparison to our pipeline. Proteogenomics Galaxy workflows [49, 50] are added at the end of the table

Another crucial step mentioned into the review by A. Nesvizhskii [7] is the confidence degree for results. Validation and cura\tion steps are not always integrated into existing proteogenomics pipelines. Peptimapper provides results annotated with quality criteria (e.g. minimal number of typical spectra by cluster) and visualizable through a genome browser for manual inspection purposes.

The high number of data processing steps that compose a proteogenomics analysis do not make the strategy easily workable for biologists. Especially since the most of available pipelines are only accessible through a command line interface or sometimes as a stand-alone software. Flexible and accessible Galaxy-based workflows presented Table 2, are implemented for proteogenomics analysis and well used for many projects [55–59]. Interestingly, through a Galaxy framework, Peptimapper is the only pipeline today that uses a complementary de novo approach that has been also proved to be efficient in finding new genes and in the discovery of refinement of intron/exon boundaries.

According to the important criteria we mentioned above a comparison of available pipelines is presented Table 2 based on these functionalities, i.e., database-driven for peptide identification or de novo peptide interpretation, then mapping onto the translated genomic sequence; pipeline interface; user-friendly for biologists; results curation; results visualization. By re-using and improving PepLine former modules, our pipeline extends the process by providing the users with new functionalities, thus meeting the important criteria and being as complete as possible: i) It is compatible with ion trap mass spectrometry data; ii) it allows quality annotation of results and their visualization through a genome browser; and iii) it makes the workflow easily accessible through the Galaxy framework [49, 50].

Annotation of the *Ectocarpus sp.* genome has become remarkably more accurate through the application of extensive RNA sequencing approaches and new informatics tools [60]. Similarly, the EctoGEM metabolic network has been considered to complement annotations within the *Ectocarpus sp.* genome database to support the understanding of metabolic networks in this organism [46]. The problems caused by many features of the *Ectocarpus sp.* genome (high number of introns per gene, extended 3’UTR, short intergenic regions) can be alleviated by accurate annotation through the use of the proteogenomics approach developed in this study.

RT-PCR experiments combined with transcriptomic data (available on ORCAE website) allowed us to confirm the predictions, validate two new genes (RPL22, AST), and correct one gene model (Dihydrolipoamide acetyltransferase), all corresponding to clusters obtained by our combined approach of proteomics and bioinformatics. Crossing the data generated by our bioinformatics workflow for another cluster (cluster B) with transcriptomic data allowed us to identify an alternative ATG start codon of a gene encoding a nucleoside diphosphate kinase. This finding suggests that there may be two alternative ATGs for this gene. Such a result shows that direct mapping of MS/MS data to genomic information provides a valuable approach to complement automatic annotation.

The methodological development focused on: i) workflow development and the optimization of parameters to apply it to all our ‘sub-proteome’ MS/MS datasets and ii) the search for the best qualifying criteria to sort clusters according to specific aims (e.g.*,* re-annotation, identification of small ORFs in the 3’UTR, etc.).

Parameter adjustment is based both on MS/MS spectra and genomic features. Fine-tuning appears to be an important step and configuration workflow settings are now available for organisms with gene characteristics similar to those of our test case. Here, we only focused on a few results. Indeed, many other identified clusters should be of potential biological interest. Sixteen additional clusters are currently under investigation in our laboratory by combining proteomics with new developments in transcriptomics [60]: nine potential new protein-coding genes are yet to be confirmed, and seven exonic models and one ATG model may need correcting (Table 3; Additional file 6).

**Table 3:** Additional clusters currently under investigation

Cluster ID	Contig	Strand	From	To	Strain	Tot pep	RNA data	Genetic map	Action	Identification
113	sctg_117	D	265421	281797	EC494	10	ESTs+RNAseq	LGUn	probable new gene	Esi0117_0046 similar sequence
179	sctg_136	D	16590	18641	EC494	3	ESTs+RNAseq	LG16	Esi0136_0001 model correction	Ferredoxin
750	sctg_346	D	52096	53724	EC494	3	RNAseq	LG15	Esi0346_0010 model correction	Esi0003_0041 similar sequence
1034	sctg_6	D	824608	831877	EC494	3	RNAseq	LG04	Esi0006_0137 model correction	Conserved unknown protein
1056	sctg_62	D	30800	38368	EC494	9	RNAseq	LG16	Esi0062_0006 model correction	Hypothetical protein
1072	sctg_634	D	21444	27984	EC494	3	ESTs+RNAseq	LGUn	probable new gene	none
1154	sctg_77	D	414499	420533	EC494	3	No data	LGUn	probable new gene	none
120	sctg_123	R	77652	82101	Ec32	4	RNAseq	LG08	probable new gene	none
220	sctg_150	D	399071	404913	Ec32	3	No data	LG11	probable new gene	none
777	sctg_43	R	146476	147692	Ec32	3	RNAseq	LG03	Esi0043_0035 model correction	Catalase
822	sctg_48	R	267223	267911	Ec32	5	RNAseq	LG23	Esi0048_0051 model correction	Hypothetical protein
492	sctg_253	D	215846	216917	Ec32	3	RNAseq	LGUn	probable new gene	none
567	sctg_291	R	60813	65620	Ec32	3	RNAseq	LG03	Esi0291_0011 model correction	mTERF domain-containing protein
618	sctg_310	D	63974	71773	Ec32	3	RNAseq	LGUn	probable new gene	none
697	sctg_365	R	137829	143627	Ec32	3	RNAseq	LGUn	probable new gene	none
218	sctg_87	R	471174	478804	Ec410	3	No data	LG26	probable new gene	Retrotransposon integrase-like protein

Identification of the clusters was obtained by Blast analysis. The contig and genetic map data correspond to the *Ectocarpus sp.* v1 genome annotation, showing supercontigs (sctg) and linkage groups (LG), respectively. Strain refers to the *Ectocarpus sp.* strain that was the origin of the protein samples. Action refers to the proposed correction of the current gene annotation according to the newly incorporated RNA data in the browser (RNA sequencing and ESTs) see Additional file 6

### Future studies

Recently, extensive RNA-seq data were used to improve 11,108 existing gene models and identify 2030 new *Ectocarpus sp.* protein-coding genes [60]. New data available in public databases has advanced functional annotation associated with protein-coding genes. To date, 61% of genes now have a functional assignment, compared to 34% in the V1 annotation [60] we used in our workflow. We are now applying our workflow, tailored for this organism, using the most recent *Ectocarpus sp.* genome annotation (http://bioinformatics.psb.ugent.be/orcae/overview/EctsiV2). In the future, we will analyze short ORFs, focusing on small clusters corresponding to gene models ≤ 150 nucleotides. In such a study, the proteogenomics approach is a clear asset to confirm whether some small transcripts are translated.

We also successfully tested Peptimapper using MS data produced by a mass spectrometer of the latest generation (i.e.*,* Q Exactive™ HF, ThermoFisher Scientific), currently used to analyze another organism (i.e., *Homo sapiens)* and other MS data analysis software (i.e., Mascot Distiller V2.6; software supported by a more recent version of the Mascot server v2.5.1; http://www.matrixscience.com) to create MGF. This shows that Peptimapper is fully adaptable to the most recent MS instruments and MS analysis software and is relevant to study other eukaryotic organisms.

## Conclusions

In addition to improving annotation of the *Ectocarpus sp.* genome and gaining new knowledge about its proteome, our objective was to provide an accessible, efficient, and flexible tool to the annotation community that is easily configurable according to the species of interest. Thus, genome sequence and GFF3 files must be available for the organism of interest to display genome features. The workflow is available as a Docker image or interfaced with our Galaxy platform (see Additional file 5), enabling web access to users with non-programming experience to easily run it in a transparent and reproducible way.

## Availability and requirements

Project name: Ectoline

Project home page: https://github.com/laeticlo/Ectoline

Operating system(s): this distribution contains sources and pre-compiled binaries for Linux, and MacOSX platform

Licence: GPL license or under the CECILL licence

Ectoline Docker image name: peptimapper

Docker hub repository: http://hub.docker.com/r/dockerprotim/peptimapper/

Docker bioshadock repository: https://docker-ui.genouest.org/app/#/container/dockerprotim/peptimapper

Galaxy platform: https://galaxy.protim.eu/

## Additional files

Additional file 1: Sample preparation protocols. (PDF 93 kb)

Additional file 2: All reference datasets Excel file. (XLS 21262 kb)

Additional file 3: All cluster results Excel file. (XLSX 282 kb)

Additional file 4: Scripts detailed descriptions: command line arguments, output file descriptions and availability. (PDF 161 kb)

Additional file 5: Bioinformatic tools distribution. **A**. Peptimapper dockerfile. **B**. Workflow labeled “Peptimapper” available on Protim Galaxy platform. (PDF 773 kb)

Additional file 6: Study of additional clusters under investigation listed Table 3. RNA-sequencing and EST data have been incorporated in the browser. (PDF 2631 kb)

## Abbreviations

CDS: Coding DNA sequence
CF: Cytoplasmic-proteome fractions
CHAPS: 3-[(3-cholamidopropyl)dimethylammonio]-1-propanesulfonate
CPU: Central processing unit
CW: Cell wall
CWF: Cell-wall-enriched proteome fractions
DTT: Dithiothreitol
EDTA: Ethylene-diamine-tetraacetic acid
EST: Expression sequence tag
FACS: Fluorescence-activated cell sorting
FDR: False Discovery Rate
GF: Gametes proteome fractions
GFF/GFF3: General feature format
HEPES: 4-(2-hydoxyethyl)-1-piperazine ethane sulfonic acid
HPLC: High-performance liquid chromatography
LC-MS/MS: Liquid chromatography tamdem mass spectrometry
MF: Membrane-enriched proteome fractions
MGF: Mascot Generic File
MS: Mass spectrometry
NES: Nuclear export signal
NF: Nuclear proteome fraction
NLS: Nuclear localization signal
PCR: Polymerase chain reaction
PST: Peptide sequence tag
RAM: Random access memory
SAAV: Single amino acid variant
SP: Secretory pathway
