# Supplementary material for: Peptimapper: proteogenomics workflow for the expert annotation of eukaryotic genomes
Source: BMC Genomics. 2019 Jan 17;20:56. doi: 10.1186/s12864-019-5431-9 (PMC6337836; doi:10.1186/s12864-019-5431-9)
Supplement: Supplementary file 1 — Sample preparation protocols. (PDF 93 kb) [file 12864_2019_5431_MOESM1_ESM.pdf]

Additional file 01: Sample preparation protocols

Algal material and culture

Ectocarpus sp. (Ectocarpales, Phaeophyceae) strains used in this study derived from the genome-sequenced Ec32 ecotype (CCAP accession 1310/4, origin San Juan de Marcona, Peru 15°22'S, 75°10'W). In addition to Ec32 (wild-type male), the Ec410 was analyzed as female strain. The life stage generation mutant strain Ec494 *ouroboros (oro)* was employed as biological material for the gametophytic stage [24]. All strains were cultivated in sterile 10 L tanks supplemented with 0.2 µm filtered air bubbling, at 14°C under 20 µE of white light at 12 h light: 12 h dark cycles. Culture medium consisted in 0.45 µm filtered then autoclaved seawater, supplemented with half-strength Provasoli medium [25].

Subproteome preparation

Isolation of cytoplasmic- (CF) and membrane-enriched (MF) proteome fractions

Sporophyte cultures of Ec 32 were used as starting material to sequentially extract cytoplasmic (CF)- and membrane-enriched (MF) proteome fractions. A total of 1g dry weight of starting material was ground under liquid nitrogen. The material was further extracted three times with 10 mL of 100 mM Tris-HCl pH 7.5, then incubated for 10 min at 4°C under slow shaking and centrifuged for 5 min at 12 000 g. The supernatants constituted the cytoplasmic proteome fractions (CF) while pellets constituted the membrane-enriched proteome fractions (MF), respectively.

The CF was concentrated by precipitation with 2 volumes of cold acetone, incubated for 1 h at -20°C then centrifuged at 10 000 g for 10 min at 4°C. The obtained pellet was washed with 80% cold acetone and solubilized in 300 µL of TL buffer containing 6 M urea, 2 M thiourea, 1% (w/v) DTT, 4% (w/v) CHAPS and Complete Protease Inhibitor Cocktail (Roche, Boulogne-

Billancourt, France). In parallel, MF was extracted from the residual pellet with 5 mL of a buffer composed of 100 mM Tris-HCl pH 7.5; 1.5 M NaCl; 50 mM DTT and 0.5% (v/v) NP40.

Isolation of cell wall-enriched proteome fractions (CWF)

A non-destructive method was employed to extract the cell wall-enriched proteome fraction (CWF) according to a protocol previously established for land plants [26]. A total of 3 g fresh tissue of Ec 32 sporophyte culture was successively extracted with four buffers consisting in (i) 15mL of 0.2 M $CaCl_2$, (ii) 2 mM DTT, (iii) 1 M NaCl and (iv) 0.2 M sodium borate. Proteins were then extracted with each buffer through incubations during 30 min at 4°C under gentle shaking. In between buffer incubations, tissue was washed twice with 5 mL of sterile seawater. All extracts were pooled and concentrated by reverse osmosis in a dialysis bag with a 3.5 kDa cutoff size (Spectrum Labs, Breda, The Netherlands) and covered with PEG 8000 to absorb the excess of liquid and salts. After concentration, the remaining 5 mL was ultrafiltered to 500 µL on a 5 kDa Centricon device (Millipore, Molsheim, France). The obtained extract was purified using the 2D clean-up kit (Bio-Rad, Marnes-la-coquette, France) then resuspended in the same volume of TL buffer and stored at -80°C until use.

Isolation of nuclear proteome fraction (NF)

A total of 10 g of Ec32 sporophytes and 16 g of Ec494 gametophytes were employed to isolate nuclear proteome fractions (NF). First, protoplasts were isolated from fresh tissue as previously described [27]. Nuclei were then isolated from protoplasts using a previously described method with some modifications [28]. A nuclear isolation buffer (NIB) composed of 30 mM $MgCl_2$; 20 mM trisodium citrate; 120 mM sorbitol; 55 mM HEPES pH 7.5; 5 mM EDTA, 0.1%; (v/v) Triton X-100; 5 mM sodium bisulfite; Complete Protease Inhibitor Cocktail (Roche) and 1:5000 SYBR®Green (Invitrogen, Cergy Pontoise, France) was used. Briefly, 2 mL of NIB was

added to 50 μL of protoplast suspension, then centrifuged at 800 rpm for 15 min at 4°C. The obtained pellet was then filtered by gravity on a 10 μM mesh and nuclei were immediately sorted using a FACS Aria flow cytometer (BD Biosciences, Le Pont de Claix, France). FACS parameters were set as follows: pressure (P) = 70 psi, vibrating frequency (f) = 90 MHz, electric field (U) = 6000 V and excitation wavelength = 488 nm. About 350 000 nuclei could be isolated per sample. Nuclei were then solubilized in 20 μL of TL buffer. NF was finally purified using the 2D clean-up kit (Bio-Rad), resuspended in the 20 μL of TL buffer and stored at -80°C until use.

Isolation of total proteins from gametes (GF)

A total of 250 mg fresh weight of female gametes from the Ec410 strain was extracted with 40 μL of a lysis buffer composed of 2% 2-mercaptoethanol and 5% sodium dodecyl sulfate in milliQ water. Sample lysis was achieved through sonication by applying 2 x 10 pulses of 50% amplitude and 25 kHz. Extracts were centrifuged for 5 min at 12 000 g, and the resulting supernatant was collected as first fraction. The remaining pellet was re-extracted with 20 μL of TL buffer and centrifuged for 5 min at 12 000 g. The supernatant was collected as the second fraction. Both the first and second fractions were finally pooled to constitute the GF and stored at -80°C until use.

Isolation of total proteins from gametophytes

650 mg of *Ectocarpus* gametophyte Ec494 were grinded in liquid nitrogen. The powder was extracted as described previously [29]. Finally, the pellet was resuspended in 500 μL of TL buffer and stored at -80°C until use.

Protein assay

The Bradford method was used to evaluate the protein concentration of the subcellular fractions according to the BioRad protein assay protocol. A calibration curve was performed with a range of 1-20 µg/mL standard bovine serum albumin.
