# Supplementary material for: Peptimapper: proteogenomics workflow for the expert annotation of eukaryotic genomes
Source: BMC Genomics. 2019 Jan 17;20:56. doi: 10.1186/s12864-019-5431-9 (PMC6337836; doi:10.1186/s12864-019-5431-9)
Supplement: Supplementary file 4 — Scripts detailed descriptions: command line arguments, output file descriptions and availability. (PDF 161 kb) [file 12864_2019_5431_MOESM4_ESM.pdf]

Peptimapper workflow Version 1.0

Peptimapper workflow has to:
1) confirm genes predictions;
2) annotate new CDS;
3) correct intron-exon junctions.

First, Peptide Sequence Tags (PST) are generated from MS/MS spectra analysis (MGF files). A PST is defined as a short peptide sequence (3 to 5 amino acids) flanked by two masses corresponding to the two adjacent polypeptides. We align PSTs in the six reading frames. A hit is the location of a PST on the sequence. Hits are clustered according to parameters. Clusters obtained are evaluated and compared with the genes predictions produced by the conventional genome annotation.

Peptimapper is a workflow composed of 4 binaries detailed below.
Note that all programs take an optional -h argument, providing help on program usage

-1- LXRunPepNovo

It takes spectra file(s) as input (dta, mgf or mzXML files) and provides a PSTs file as output.
It runs PepNovo_bin (https://github.com/jmchilton/pepnovo) with default options: -model CID_IT_TRYP and -PTMs C+57:M+16 and converts output format to be compatible with PMMatch.

Running LXRunPepNovo and command line arguments:

```
-----------------------------------------------------------------------
# usage: LXRunPepNovo [-v] [-h] [-t taglen] [-n numsol] mgf_or_pkl_files+
# usage: -v : verbose
# usage: -h : print this help and exit
# usage: -t : set tag length (default 3) (only lengths 3-6 are allowed)
# usage: -n : set max number of solutions (default 10)
-----------------------------------------------------------------------
```

Output tags file format:

```
# Tags generated by PepNovo+
SpectrumTITLE.PSTId NTermMass CTermMass Tag ParentMass Score
...
%EndOfTags
```

...repeat for each tag

SpectrumTITLE: MGF file spectrum "TITLE=" value
PSTId: spectrumId '.' pstIndex where pstIndex goes from 1 to number of PSTs generated for this spectrum
NTermMass: mass of the NTerm part of the PST
CTermMass: mass of the CTerm part of the PST
Tag: PST Tag
ParentMass: mass of the Parent Ion (M+H)
Score: PST score

-2- LXRunPepMatch

It takes PST file(s) and Fasta genome sequence as input and provides a hits file and a clusters file as output.

LXRunPepMatch launches successively 3 scripts:

- PMTrans: translates a DNA file (Fasta format) into six-frame translations.
- PMMatch: matches PSTs found by LXRunPepNovo on the six-frame translation of DNA sequence. A hit corresponds to the location of a PST on a translated sequence.

Default options: -n 1 (maximum number of modified residues), -m 0 (maximum number of miscleavage(s)), -a /EctoLine/scripts/data/aa_mono.ref (amino acid reference file), -e /EctoLine/scripts/data/trypsine.ref (digestion enzyme reference file).

- PMClust: clusters hits into clusters and projects hits position back to the original (untranslated) DNA sequence.

Running LXRunPepMatch and command line arguments:

```
-----------------------------------------------------------------------
# usage: LXRunPepMatch [-v] [-h] [-f] [-F] [-D tolerance] [-t minhit] [-T minpep] [-d dist] chromo_fasta tag_file+
# usage: -v : verbose
# usage: -h : print this help and exit
# usage: -f : force recompute chromosome translation
# usage: -F : force recompute PMMatch
# usage: -D : set mass tolerance (default 0.5)
# usage: -t : set min number of hit per cluster (default 3)
# usage: -T : set min number of peptide per cluster (default 2)
# usage: -d : set clustering distance (default 5000)
-----------------------------------------------------------------------
```

Output Hits file description:

SequenceName D|R frame SeqLength (Sequence Comment)
PST_output_line_from_PepNovo_bin
F|N|C posMn posMc posTn posTc pepSeq pepMatch nbMis nbOver mbMod score
%EndOfHits
...repeat for each hit

SequenceName: name of DNA sequence (from fasta file)
D|R: D for direct strand R for reverse strand
frame: translation frame (1, 2 or 3)
SeqLength: length of original DNA sequence
PST_output_line_from_PepNovo_bin : ***PSTId NTermMass CTermMass Tag ParentMass Score***, see PepNovo_bin output format.
F|N|C: Type of hit:

- ***F:*** Full hit, both N and C term masses match
- ***N:*** N_Partial hit, C term mass is missing
- ***C:*** C_Partial hit, N term mass is missing

posMn: position of the N term part of PST on the proteic sequence (see figure below)
posMc: position of the C term part of PST on the proteic sequence (see figure below)
posTn: start position of the tag part of PST on the proteic sequence (see figure below)
posTC: end position of the tag part of PST on the proteic sequence (see figure below)

```
    (Mn)-----------[ tag ]----------(Mc)
      |            |      |           |
      |            |      |           |
  ___|__________|_____|________|___
   posMn     posTn posTc   posMc
```

Note: all positions are given on the translated sequence
Note: for partial hits (N or C types), the missing ***posMx*** position is indicated by 0
pepSeq: the matched peptide as it appears on the translated sequence.
Note: pepSeq is flanked by the preceding and following symbol on the sequence:
<preceding>.peptide.<following>
special flanking symbols are '*' for sequence extremities and 'X' for a stop codon.
pepMatch: the matched peptide as matched by PMMatch, i.e. with possible modifications. if present, modified amino-acids are indicated by lowercase characters. Moreover, the tag part of the PST is indicated between brackets.
nbMis,nbOver : number of mis- and over-cleavages. Normally, a hit should be flanked by two enzymes cut sites or a stop codon on the C-Term or a Met on a N-Term and no cut sites should appear within the peptide. An extra cut site within the peptide is called a mis-cleavage. A missing cut site at any extremity is called an over-cleavage.
Note: the maximum number of mis- and over-cleavage can be set by the -m and -M options.
Note: a stop codon at the C-term part is not counted as an overcleavage
Note: a Methionine at the N-term part is not counted as an over-cleavage unless the -y option is set to 0.
nbMod: number of modified aa in the peptide.
Note: allowed modifications are indicated in the 'aa_mono.ref' runtime configuration file (see below).
Note: the maximum number of modified aa can be set with the -n option

Note: in the current version, no modifications are allowed in the tag part of the PST, only modifications in the N or C terminal masses are taken into account.
score: PST score.

Output Clusters file description:
CLUSTER idCluster nbHits nbFullHits nbPepHits score
SequenceName 'D|R' frame SeqLength (Sequence Comment)
// the two following lines are rpeated for each hit in the cluster
PST_output_line_from_PepNovo_bin
HIT_output_line_from_PMMatch
%EndOfCluster
...repeat for each cluster

idCluster: an integer starting at 1
nbHits: total number of hits (full + partials)
nbFullHits: number of full hits
nbPepHits: number of different peptides
score: score of cluster (in the current version the sum of full hits scores)
SequenceName: name of DNA sequence (from fasta file)
D|R: ***D*** for direct strand ***R*** for reverse strand
frame: translation frame (1, 2 or 3)
SeqLength: length of original DNA sequence
Note: this line is identical to the sequence line in the hit output of PMMatch.

!!! Important note: the ***posMn, posMc, posTn, posTc*** positions are now given as nucleotide positions on the original DNA sequence.

-3- LXQualify

It takes clusters file(s) and GFF directory as input and provides annotated or unannotated clusters file as output

Running LXQualify and command line arguments:

```
---------------------------------------------------------------------------
#usage: LXQualify [-v] [-h] [-f] cluster_file gff_directory
#usage: -v : verbose
#usage: -h : print this help and exit
#usage: -f : force recompute chromosome translation
---------------------------------------------------------------------------
```

-4- LXClust2Gff

It processes all cluster files, and produce GFF files for each clusters file to be integrated into a genome viewer.
Running LXClust2Gff and command line arguments:

```
---------------------------------------------------------------------------
# usage: LXClust2Gff [-v] [-h] gff_directory output_directory cluster_file+
# usage: -v : verbose
# usage: -h : print this help and exit
---------------------------------------------------------------------------
```

Availability

- Sources and pre-compiled binaries for Linux and MacOS platforms are available at https://github.com/laeticlo/Ectoline.
- A Docker image, peptimapper (https://docker-ui.genouest.org/app/ - /container/dockerprotim/peptimapper) allow easier distribution and interoperability of scripts.
- Scripts for each step of the overall workflow were integrated and deployed on a Galaxy server https://galaxy.protim.eu), enabling web access to users with non-programming experience to easily run the workflow in a transparent and reproducible way.

For any information about Peptimapper, please send an email to : laetitia.guillot@univ-rennes1.fr
