# Supplementary material for: Peptimapper: proteogenomics workflow for the expert annotation of eukaryotic genomes
Source: BMC Genomics. 2019 Jan 17;20:56. doi: 10.1186/s12864-019-5431-9 (PMC6337836; doi:10.1186/s12864-019-5431-9)
Supplement: Supplementary file 5 — Bioinformatic tools distribution. A. Peptimapper dockerfile. B. Workflow labeled “Peptimapper” available on Protim Galaxy platform. (PDF 773 kb) [file 12864_2019_5431_MOESM5_ESM.pdf]

A.

```
FROM centos:centos7

# name of the maintainer of this image
MAINTAINER laetitia.guillot@univ-rennes1.fr
USER root
# install the latest upgrades
RUN yum -y update && yum clean all
RUN yum -y install csh
RUN yum -y install unzip
RUN yum -y install zip
# install dos2unix et git
RUN yum -y install git git-core git-daemon-run
RUN yum -y install dos2unix

RUN git clone https://github.com/laeticlo/Ectoline.git EctoLine
RUN dos2unix /EctoLine/thirdparty/pepnovo/pepnovo.3.1b/Models/*.*
RUN dos2unix /EctoLine/thirdparty/pepnovo/pepnovo.3.1b/Models/*/*.*

ENV PATH /EctoLine/ports/x386-linux/bin:$PATH
ENV PATH /EctoLine/scripts:$PATH
```

B.

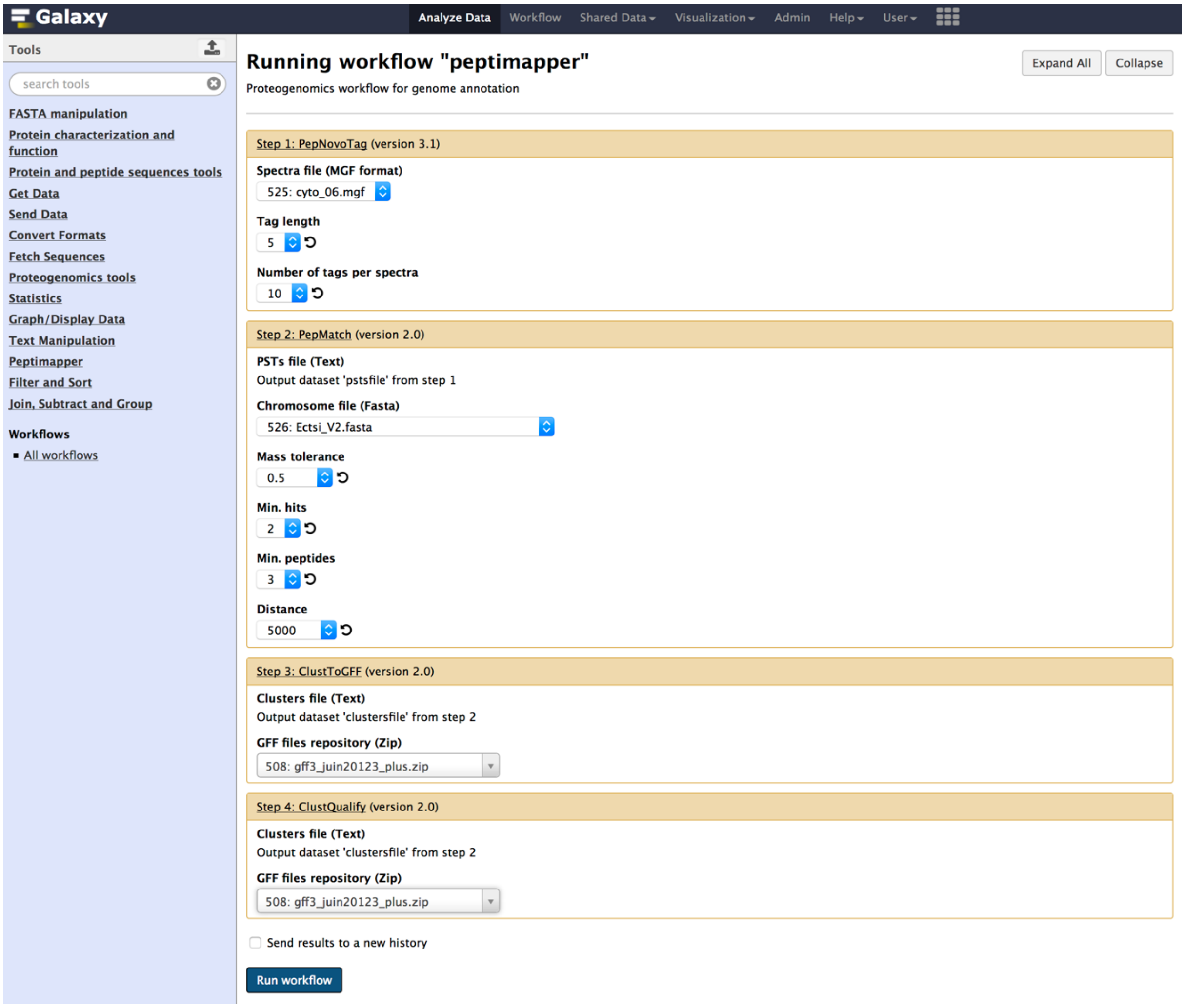

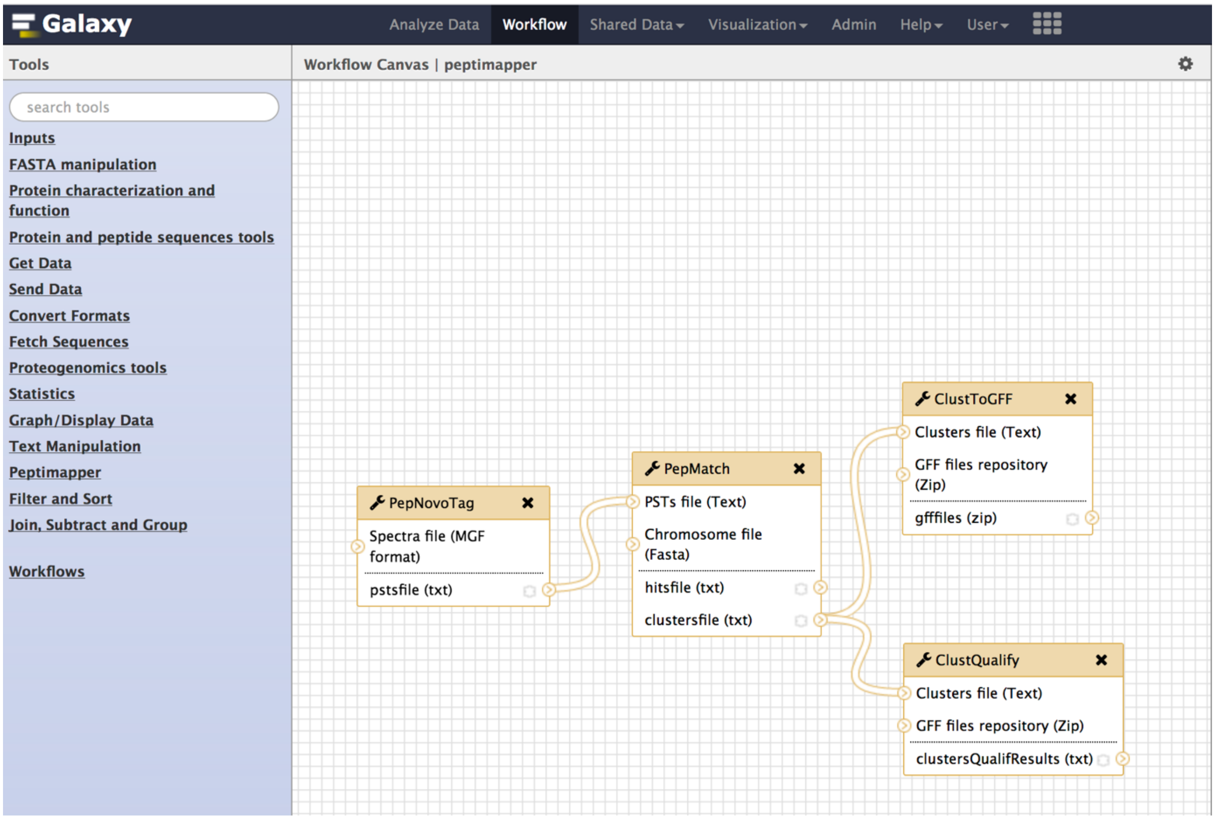
Galaxy
Analyze Data
Workflow
Shared Data
Visualization
Admin
Help
User
Tools
Workflow Canvas | peptimapper
search tools
Inputs
FASTA manipulation
Protein characterization and function
Protein and peptide sequences tools
Get Data
Send Data
Convert Formats
Fetch Sequences
Proteogenomics tools
Statistics
Graph/Display Data
Text Manipulation
Peptimapper
Filter and Sort
Join, Subtract and Group
Workflows
PepNovoTag
Spectra file (MGF format)
pstsfile (txt)
PepMatch
PSTs file (Text)
Chromosome file (Fasta)
hitsfile (txt)
clustersfile (txt)
ClustToGFF
Clusters file (Text)
GFF files repository (Zip)
gfffiles (zip)
ClustQualify
Clusters file (Text)
GFF files repository (Zip)
clustersQualifResults (txt)
