# Supplementary material for: Peptimapper: proteogenomics workflow for the expert annotation of eukaryotic genomes
Source: BMC Genomics. 2019 Jan 17;20:56. doi: 10.1186/s12864-019-5431-9 (PMC6337836; doi:10.1186/s12864-019-5431-9)
Supplement: Supplementary file 6 — Study of additional clusters under investigation listed Table 3. RNA-sequencing and EST data have been incorporated in the browser. (PDF 2631 kb) [file 12864_2019_5431_MOESM6_ESM.pdf]

Guillot et al., Additional File 06

This file provides information on the methology of analysis of each PST cluster presented in the Table 2. Following are representation of the genome view for the corresponding zone extracted from the ORCAE database (http://bioinformatics.psb.ugent.be/orcae/overview/Ectsi).

The cDNA (CDS) model of a gene is presented when it exists together with the exonic (in blue or green) and the intronic (in pink) zones given by RNA-Seq data and EST data (in light green).

For all of the clusters described in Table 2 no RT-PCR validation was done, therefore conclusions are only based on transcriptomic data.

When possible we proposed a new CDS model for the corresponding gene or encoded protein evidence based on EST sequences. In the article, we have suggested the nature of the possible reannotation (new gene or gene model correction) as a conclusion.

CLUSTER 113
sctg_117 : 265421..281797

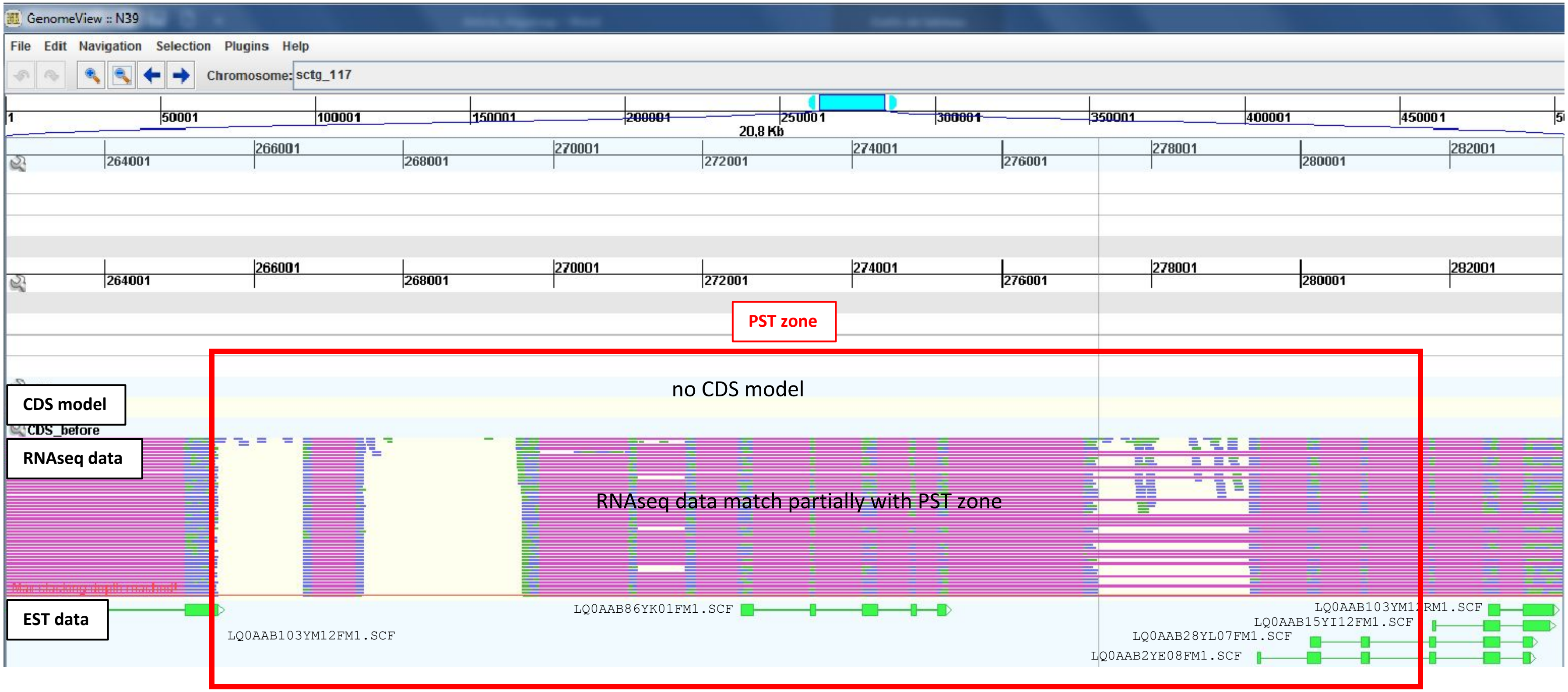

6 ESTs match with RNAseq data and PST zone

RT-PCR data No RT-PCR experiment was conducted for this cluster

CLUSTER 113

sctg_117 : 265421..281797

ESTs sequences:
>LQ0AAB103YM12FM1.SCF
GAGAGTGAAAACCGTGCAGAGGGTGCAACAACCAAGGTTGCGGCCTCTTTCTCAACCGCTCCGGCATCACCGGAACTTGGAAGCGGTTGAGCTGGTGGGAGACAGCTATAT
CTGCTCGTGTTGCGGAGCCACAAGGGCGCCTTGGCAGGACTTTCGGTGAAGAGCGTTACCCGACAGGAGGTAGCAGAGAACCATGGCGGCGGACACGACAACGACAACGGA
TACGACGGCAGAGACGACAACAACCACCGTCGATGCCACCCAAGTACTGACCGACTTCAGCGAGGTCGTCGCCTCGCTCAAGGCTTCCTGCGAAGCAGACGTGACCACGGC
CGCCCTGTCCTCGGTGATGGGAAAGCTCAAGATCATGGCCGATGCCGTCGCCGCTCTGGACGCCTCCCCGACTCTCAAGAGCATGCAGCTCAGCTCCGACATGGCCGAGCT
GGCGCTGCTCTGCCTCCCACCCGACACGAAGGACGTGCTGCTGGGCACCGGCTGCTCGAGCGACCTGGCTCTGGAGCTCGTATGCCGCTACCTCACCAAGCTGCCGGCCCT
CGCAAAGACTCTGTACAAG
>LQ0AAB86YK01FM1.SCF
TTGGGAGCAGTCCATGGCTTCCAACTTCACCAAGACCACGGAGAACGGGGGCATCAGCATCGCACAGTCCTCCTCCACGTCCTGGGGCGTCAGCGCCAGCGTCTCGTCCTT
CGGGGGCGCCGCGAGCGCGAGCGTTTCGGCGGACTCGAGCGGGACGGAGGAAGGGACCGGAGAAGCCAAGGGGAGCAACGCCTTCTCCGCGAACAACAAGACCACCACCAC
GTCGGCGACCACCGTGGAGACCATCACGTGCCCCATGAAGTCGTTCCGCATCCCGGTGTCCACCATGACGATGACGGAAGAGGCTCTCGACCACGCCTTGGAGGTGGACAC
CCTGCTCAAGGCCGGGCAGTTCCTCGACACCTTCGGCTCCCACGTCTCCAACGGCCGGCACCTCCTCGGGGGAATTTTCTTCCGCACCATGACCATGACCACGGAATCCGA
GGTGGACACCTCCACCCTCCTCGCCGCCGCCAGCACCCAGATGCAGCAGAGCAGGACCGAGTCCAGCGACTCCACTGCCTCAGCGGGCGCCGGGACCGTGGGTGTCTCTGT
GGCTGCGAGTGCCACCATGAACAGCGCCTCCAGCTCGGGAGTCGTCAAGTTTCACGCCACCGCCGGCCACAGCGAGAGCGAGTC
>LQ0AAB28YL07FM1.SCF
TTGGGGAGTCGAAGCCTCTCGGACAGGCCGATTTCTTCCCGACGCTGCAGTCCAAGATAGCCGAAACTCCCGAGATCACCGGAAAGTACGACTTCAAGACGAACACGACCG
AGTACCAGACCGCGGCGAGGAGCGCGAAGAAGCTGTCGTCGACGGATTGGGTGGGGTTCGACCTCATCTGGAAGAAGCGTCTCGGCAACATCGCTCTCAAGGAAGCCCTCG
AGACCCACATCAAGTTCGAAGAGGCGACGCTGAGGCTTGGCGAGGTCATCACCACCGATGAGCCGCTCACCAGCGTCGACGGGAACTACCGTCTCGAGATATCTGGAAAGG
CGCTGAGGGTCGTCAAGACTAGCACCGGCGCCGTAGCTGCAACCCTCTTCGAGGCGGAGGGCACGCTCGGCAGCAGCGCCAGCGCGCACATGGAGGAGAACGGGTCGTTCG
TGCTGGACCTCACCATGATCACGGGCTTCGATCGCACGGAAACCCGGATGGTTCTCTGGGTCGCGCCCAACACGGAGTCCGGCAGCGGCGGCTACGCCAGGATGCAAAACA
ATGGAAAGTTCGTCGTTTTCAACCGGAAGGGCAAGGCGCTGTGGTCCGCTGCTTCTTGAGGAAAACCCCGGGGGCATCGTTCGTTGTCGTCGTC
>LQ0AAB15YI12FM1.SCF
GAAGTCGGCGATGAGCCGCTCATCAGCGTCGACGGGAACTACCGTCTCGAGATATCTGGAAAGGCGCTGAGGGTCGTCAAGACTAGCACCGGCGCCGTAGCTGCAACCCTC
TTCGAGGCGGAGGGCACGCTCGGCAGCAGCGCCAGCGCGCACATGGAGGAGAACGGGTCGTTCGTGCTGGACCTCACCATGATCACGGGCTTCGATCGCACGGAAACCCGG
ATGGTTCTCTGGGTCGCGCCCAACACGGAGTCCGGCAGCGGCGGCTACGCCAGGATGCAAAACAATGGAAAGTTCGTCGTTTTCAACCGGAAGGGCAAGGCGCTGTGGTCC
GCTGCTTCTTGAGGAAAACCCCGGGGGCATCGTTCGTTGTCGTCGTCAACCTTGCGGGGGCGCATAGATAGCTTTCGATCCGCCCCACGCTCTCAACCGCGTTGAAGTTCC
TAGGCCGTGCCCGAGACGCCCAAACATTCCACCTGCCTACCTACCGTTTGCCGTTCACTTTTTTCGCTGCGGGGTGCGCCCACTGCTGCTTGCTTTGGACGGTAGGTAGTT
TTGTGTGCTAAACAGCGGTTTCTTTTGATACTAAGCCGACCTGCGAGGCTGTTGAAGGTTGGTTAATAGG
>LQ0AAB103YM12RM1.SCF
CAGGTTAAAAAAAAAGTATTTCAGGCAGTCTTGCCCAACCCAACCGTACCTATTACCAACCGTCAACAGCCCCCCAGGTCGGCTTAGTATCAAAAAAACCGCTGTTTAGCA
CACAAAACTACCTACCGTCCAAAGCAAGCAGCAGTGGGCCCACCCCCCAGCGAAAAAAGTGAACGGCAAACGGTAGGTAGGCAGGTGGAATGTTTGGGCGTCCCGGGCACG
GCCTAGGAACTTCAACGCGGTTGAAAGCGTGGGGCGGATCGAAAGCTATCTATGCCCCCCCGCAAGGTTGACAACAACAACAAACGATGCCCCCGGGGTTTTCCTCAAAAA
GCAGCGGACCACAGCCCCTTGCCCTTCCGGTTGAAAACGACGAACTTTCCATTGTTTTGCATCCTGGCGTACCCCCCCCTGCCGGACTCCGTGTTGGGCGCGACCCAAAAA
ACCATCCGGGTTTCCGTGCGATCAAACCCCGTGATCATGGTGAGGTCCAGCACGAACGACCCGTTCTCCTCCATGTGCGCGCTGGCGCTGCTGCCGAGCGTGCC
>LQ0AAB2YE08FM1.SCF
GAAGTTGGGTCATCAACCTGTTCGAGATCCTGGAAAGCGAGGTCGAGTCTAACTTGGAGGAGGCAGCGGAGGCCATCTCGAATGCGGTGCTCGCCACCGCCAAGGAGATGG
AGTCGAAGCCTCCCGGACAGGCCGATTTCTTCCCGACGCTGCAGTCCAAGATAGCCGAAACTCCCGAGATCACCGGAAAGTACGACTTCAAGACGAACACGACCGAGTACC
AGACCGCGGCGAGGAGCGCGAAGAAGCTGTCGTCGACGGATTGGGTGGGGTTCGACCTCATCTGGAAGAAGCGTCTCGGCAACATCGCTCTCAAAGAAGCCCTCGAGACCC
ACATCAAGTTCGAAGAGGCGACGCTGAGGCTTGGCGAGGTCATCACCACCGATGAACCGCTCACCAGCGTCGACGGGAACTACCGTCTCGAGATATCTGGAAAGGCGCTGA
GGGTCGTCAAGACTAGCACCGGCGCCGTAGCTGCAACCCTCTTCGAGGCGAAGGGCACGCTCGGCAGCAGCGCCAGCGCGCACATGGAGGAGAACGGATCGTTCGTGCTGG
ACCTCACCATGATCACGGGCTTCGATCGCACGGAAACCCGGATGGTTCTCTGGGTCGCGCCCAACACGGAGTCCGGCAGCGGCGGCTACGCCAGGATGCAAAATAATGGAA
AGTTCGTCGTTTTCAACCGGAAGGGCAAGGCGCTGTGGTCCGCTGCTTCTTGAGGAAAACC

LQ0AAB86YK01FM1 Protein sequence:
WEQSMASNFTKTTENGGISIAQSSSTSWGVSASVSSFGGAASASVSADSSGTEEGTGEAKGSNAFSANNKTTTTSATTVE
TITCPMKSFRIPVSTMTMTEEALDHALEVDTLLKAGQFLDTFGSHVSNGRHLLGGIFFRTMTMTTESEVDTSTLLAAAST
QMQQSRTESSDSTASAGAGTVGVSVAASATMNSASSSGVVKFHATAGHSESES

CONCLUSION: probable new gene → **Esi0117_0046 similar sequence**

CLUSTER 179
sctg_136 : 16590..18641

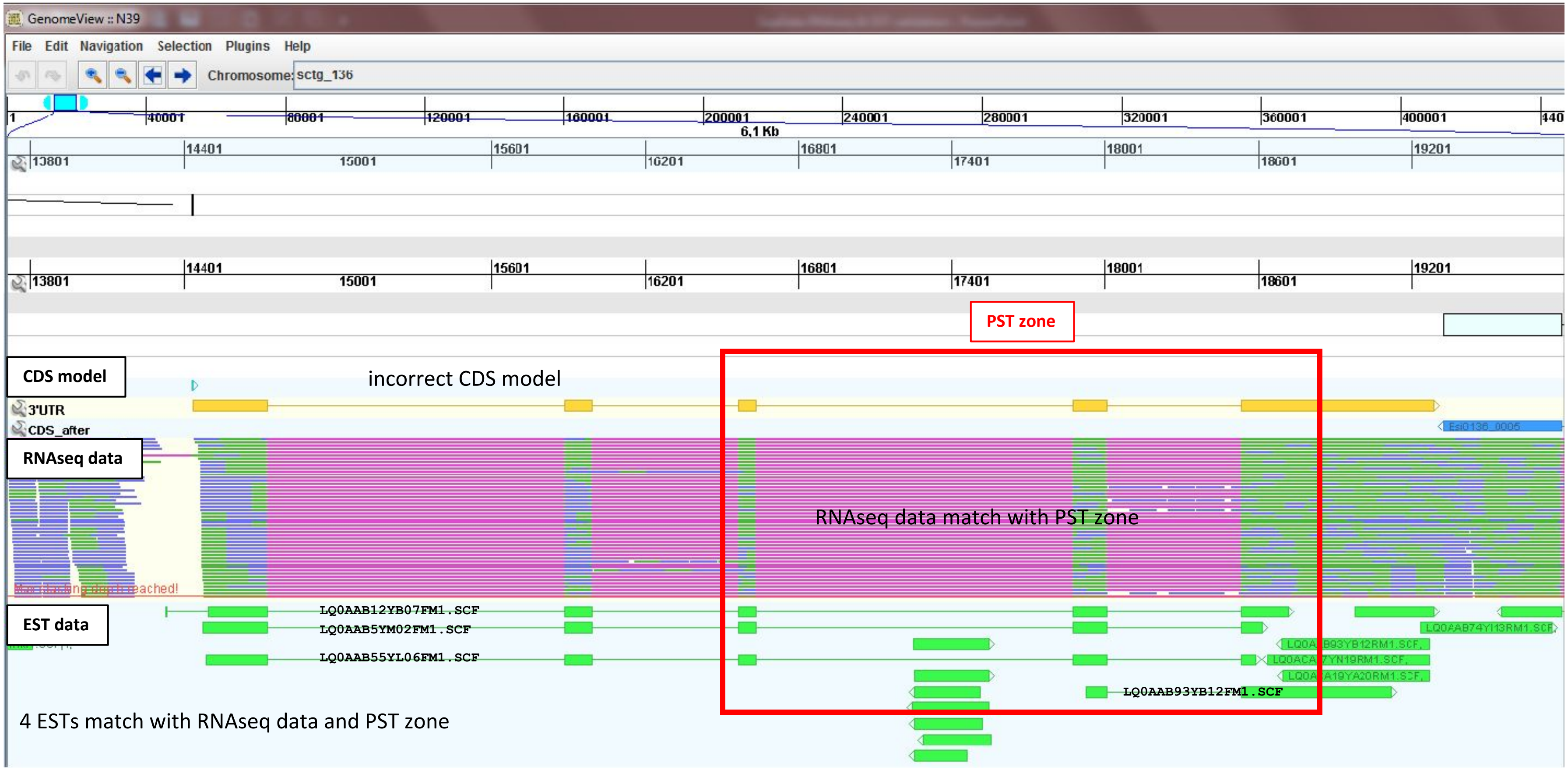

RT-PCR data

No RT-PCR experiment was conducted for this cluster

CLUSTER 179
sctg_136 : 16590..18641

ESTs sequences:
>LQ0AAB55YL06FM1.SCF
TTGGCGCGACAGCGGTCTCTCTCGAAGAGTTCCCATCCGCCAGCTACATCAAGTCTAAGGTTTCGCTCGAGAGAAGCGGG
GTTCGATTGCGAGGCTGATCGGTTTTCCAACACGCAAGGGAGCCCGCGAGAGGATCATGAAGACCAGTGCATCACATGCG
GCTGTGGCGATTGCGCTAGCCAGCGGAGTCTGCCGTTCGAGCGCGTTTGTCGTCGGTGTTGGTGGGGCGATGGCCAGGCC
TTCGGCGTCAGCGGCTAGAACAGCAGCAACGAGTCCGTTGATGGCCGTGCCTCTGGAGCTGGAGGGACAGCTCGACGAGA
GCAAGGAGTGGGAGGTGGAGCTGGAGCTCGATGGAGAGACGAAAATGGTGACAGTCCCGGAGGGCACCTCCGTCCTTTAC
GCGGCCAAGTCGGTTTTCGACGACCCGCCTTGCTCTTGCCAACAAGGGATTTGCACAACGTGTGCAGGCTTTATCAGGGA
GGGGACCAGGGACGAGAACTACAAGGTGGCCGTGAACGCGCTGGGCGAGGACCAGAGGGAAAAGGGGTTCACGCTGACCT
GCCAGACGTTCCCTTGCGGACCGGGCTTGAAGGTGTTGCT
>LQ0AAB5YM02FM1.SCF
TTGGTTTTCCTCTTCTTCTTCGCGACAGCGGTCTCTCTCGAAGAGTTCCCATCCGCCAGCTACATCAAGTCTAAGGTTTC
GCTCGAGAGAAGCGGGGTTCGATTGCGAGGCCGATCGGTTTTCCAACACGCAAGGGAGCCCGCGAGAGGATCATGAAGAC
CAGTGCATCACATGCGGCTGTGGCGATTGCGCTAGCCAGCGGAGTCTGCCGTTCGAGCGCATTTGTCGTCGGTGTTGGTG
GGGCGATGGCCAGGCCTTCGGCGTCAGCGGCTAGAACAGCAGCAACGAGTCCGTTGATGGCCGTGCCTCTGGAGCTGGAG
GGACAGCTCGACGAGAGCAAGGAGTGGGAGGTGGAGCTGGAGCTCGATGGAGAGACGAAAATGGTGACAGTCCCGGAGGG
CACCTCCGTCCTTTACGCGGCCAAGTCGGTTTTCGACGACCCGCCTTGCTCTTGCCAACAAGGGATTTGCACAACGTGTG
CAGGCTTTATCAGGGAGGGGACCAGGGACGAGAACTACAAGGTGGCCGTGAACGCGCTGGGCGAGGACCAGAGGGAAAAG
GGGTTCACGCTGACCTGCCAGACGTTCCCTTGCGGACCGGGCTTGAAGGTGTTGCTCAACCAGTACGACACCGTGTACG
>LQ0AAB12YB07FM1.SCF
TTGGAGCGGTCTCTCTCGAAGAGTTCCCATCCGCCAGCTACATCAAGTCTAAGGTTTCGCTCGAGAGAAGCGGGGTTCGA
TTGCGAGGCTGATCGGTTTTCCAACACGCAAGGGAGCCCGCGAGAGGATCATGAAGACCAGTGCATCACATGCGGCTGTG
GCGATTGCGCTAGCCAGCGGAGTCTGCCGTTCGAGCGCGTTTGTCGTCGGTGTTGGTGGGGCGATGGCCAGGCCTTCGGC
GTCAGCGGCTAGAACAGCAGCAACGAGTCCGTTGATGGCCGTGCCTCTGGAGCTGGAGGGACAGCTCGACGAGAGCAAGG
AGTGGGAGGTGGAGCTGGAGCTCGATGGAGAGACGAAAATGGTGACAGTCCCGGAGGGCACCTCCGTCCTTTACGCGGCC
AAGTCGGTTTTCGACGACCCGCCTTGCTCTTGCCAACAAGGGATTTGCACAACGTGTGCAGGCTTTATCAGGGAGGGGAC
CAGGGACGAGAACTACAAGGTGGCCGTGAACGCGCTGGGCGAGGACCAGAGGGAAAAGGGGTTCACGCTGACCTGCCAGA
CGTTCCCTTGCGGACCGGGCTTGAAGGTGTTGCTCAACCAGTACGACACCGTGTACGAGATGCAGTACGGCCAGTACGAG
GTCAAGGCCGACGAGCCCAAGAAGATGTTCGGAATTTTCTAAGAAGTTGCAAGTGTATGATTTTCCCGCGATTTTTTCTG
>LQ0AAB93YB12FM1.SCF
TTGGGGCTTTATCAGGGAGGGGACCAGGGACGAGAACTACAAGGTGGCCGTGAACGCGCTGGGCGAGGACCAGAGGGAAA
AGGGGTTCACGCTGACCTGCCAGACGTTCCCTTGCGGACCGGGCTTGAAGGTGTTGCTCAACCAGTACGACACCGTGTAC
GAGATGCAGTACGGCCAGTACGAGGTCAAGGCCGACGAGCCCAAGAAGATGTTCGGAATTTTCTAAGAAGTTGCAAGTGT
ATGATTTTCCCGCGATTTTTTCTGATTTATTATGTTCCGGAAAATTATGCGGAGAACCCGGGTAGCCGAACACAATTTTA
TTGGAAATGGGGGCCGAGAGGTTTCTGACCTCCAGAATTTTCCAAGAAAACATGTTCCCATGGCCTTTTTTTGTCTCGTT
TTAAGGCCGCTCCGGTACCTGGCAGCTGTAGTGGCGTACGGATGGAAGCACAGTCTATGAGGGCAGCGGTCGGGCACCAT
GACAAACCAAGATACAACATGTCTGATGTGCGTTGTGTGCGACCGGGCATAGAGACCGCCGATAAGACCGATCAATCCTC
TTGCTGTAGCAGGTATAGCGCTTGTCTTTCGTTGCGCTTGGTGGACGTTTACTTCCGTCTTGAGTCAGCCATCTGTTTTT
TTGTAGTTTCGTTCTTGTCTTGGCTTG

Protein sequence:
MKTSASHAAVAIALASGVCRSSAFVVGVGGAMARPSASAARTAATSPLMAVPLELEGQLDESKEWEVELELDGETKMVTV
PEGTSVLYAAKSVFDDPPCSCQQGICTTCAGFIREGTRDENYKVAVNALGEDQREKGFTLTCQTFPCGPGLKVLLNQYDT
VYEMQYGQYEVKADEPKKMFGIF*

CONCLUSION: **Esi0136_0001 model correction** → hydroxyacylglutathione hydrolase + **new gene of ferredoxin 2Fe-2S**

CLUSTER 750
sctg_346 : 52096..53724

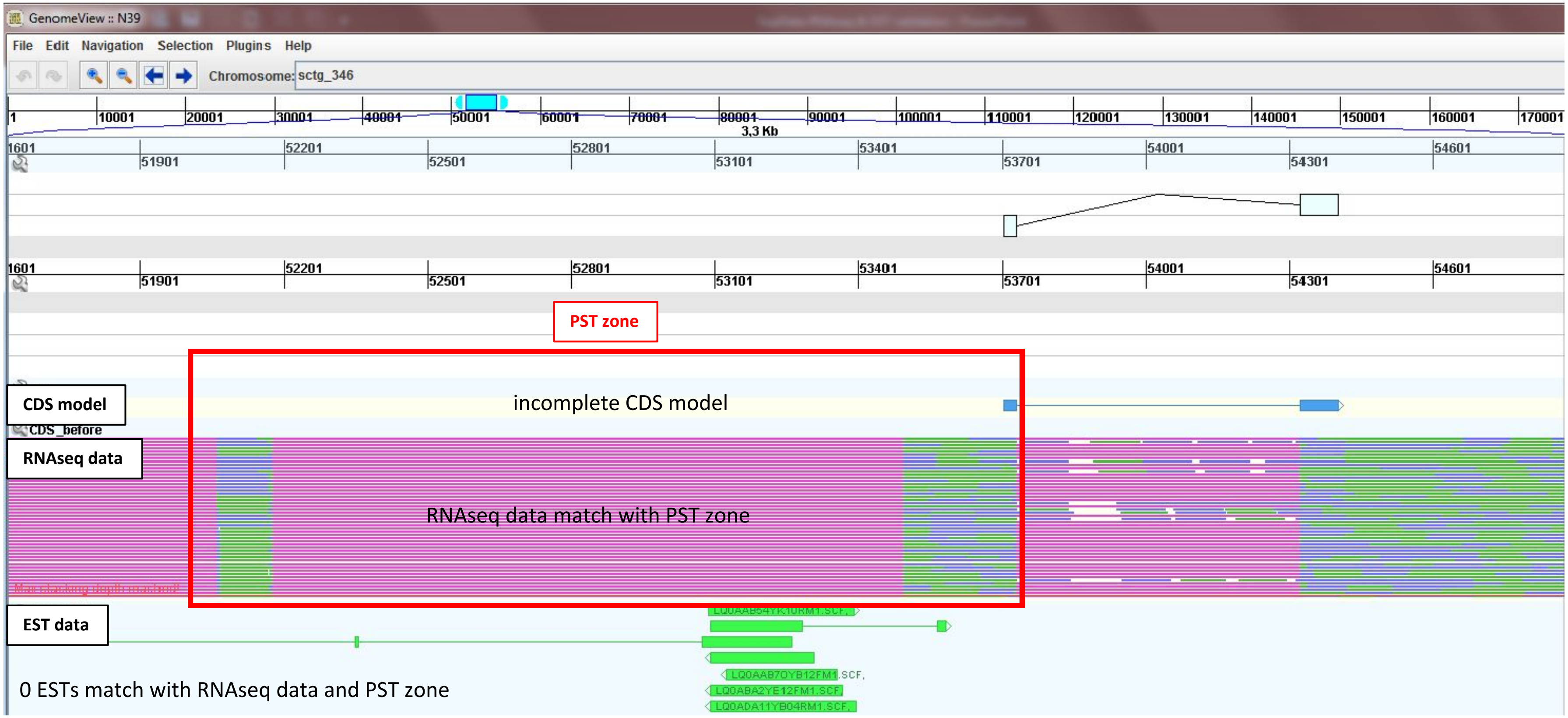

RT-PCR data No RT-PCR experiment was conducted for this cluster

CONCLUSION: **Esi0346_0010 model correction** (protein similar to Esi0003_0041 sequence)

CLUSTER 1034
sctg_6 : 824608..831877

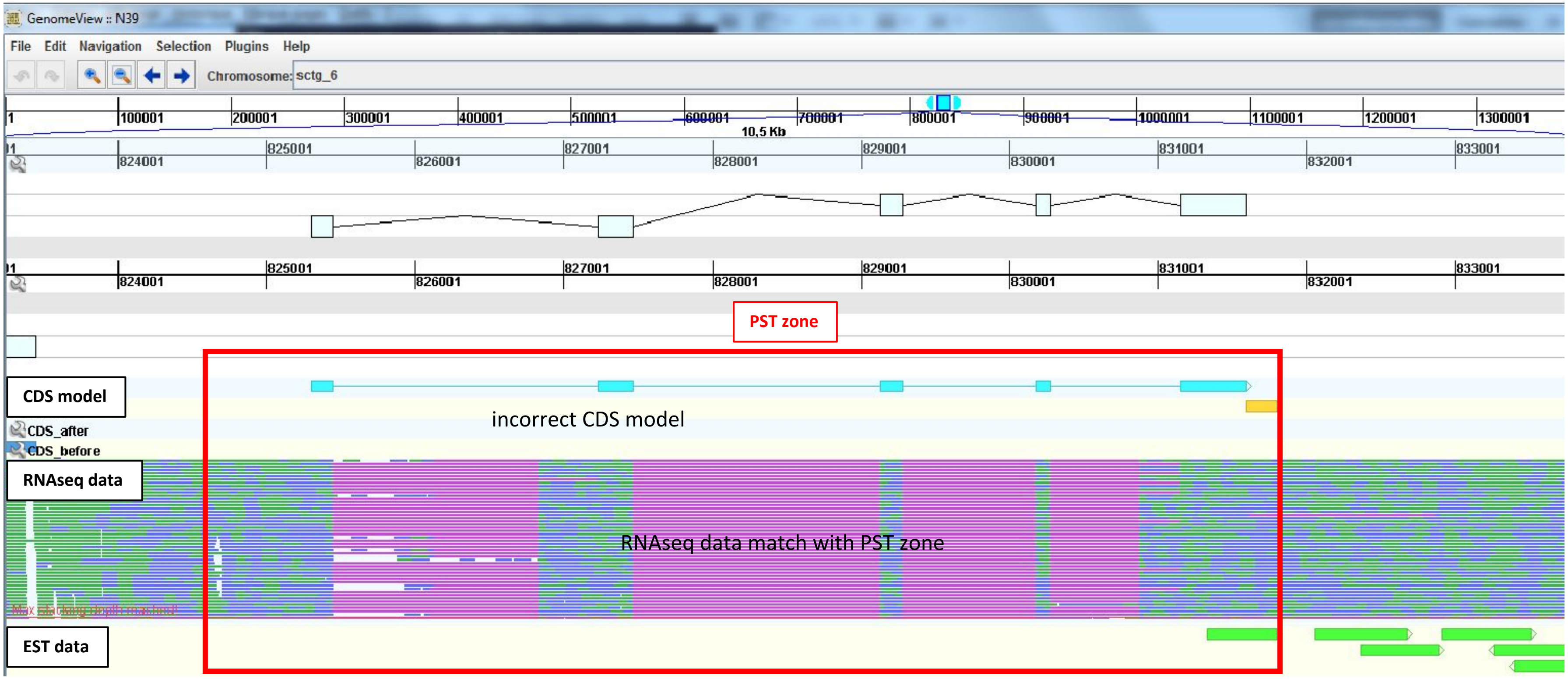

1 EST matches with RNAseq data and PST zone

RT-PCR data No RT-PCR experiment was conducted for this cluster

CONCLUSION: **Esi0006_0137 model correction** (conserved unknown protein)

CLUSTER 1056
sctg_62 : 30800..38368

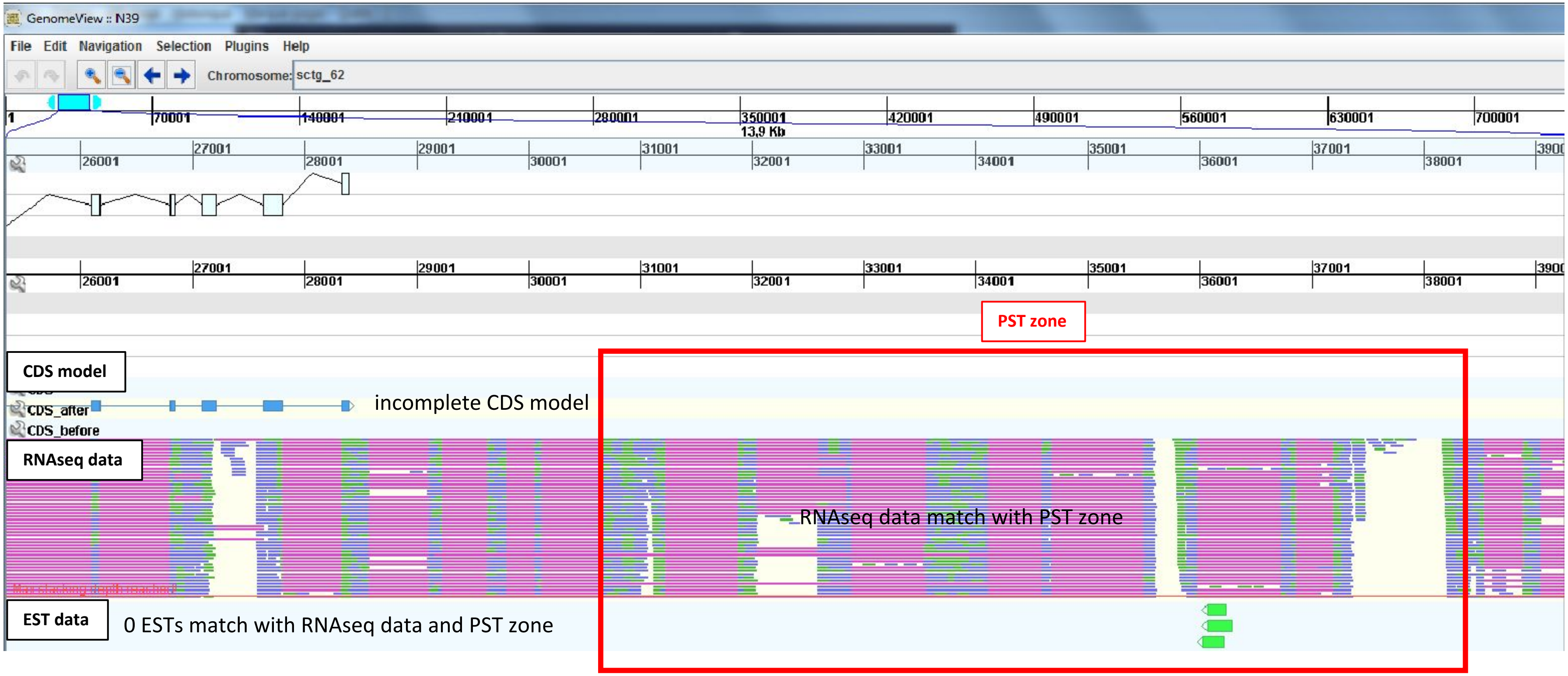

RT-PCR data

No RT-PCR experiment was conducted for this cluster

CONCLUSION: Esi0062_0006 model correction (hypothetical protein)

CLUSTER 1072
sctg_634 : 21444..27984

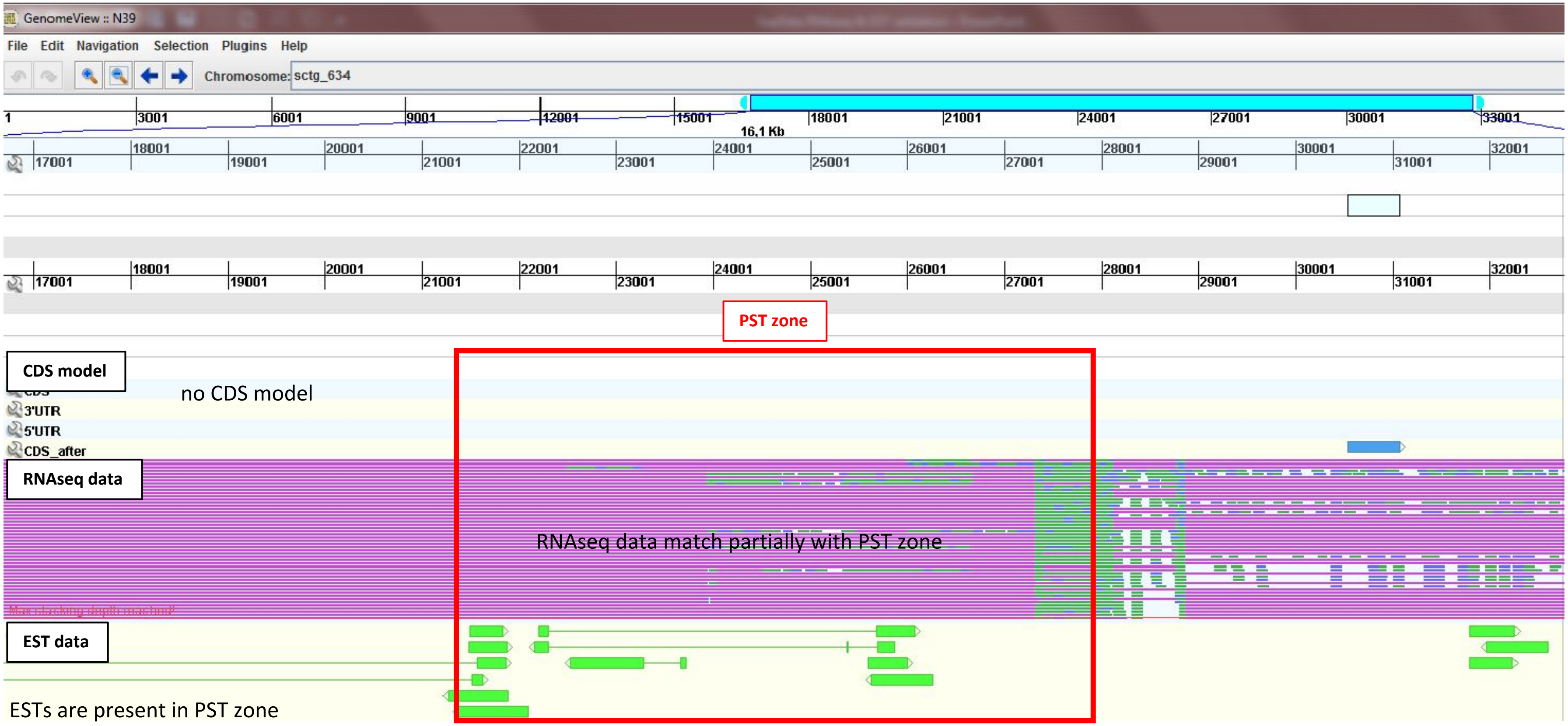

ESTs are present in PST zone
ESTs and RNAseq data do not match together

RT-PCR data

No RT-PCR experiment was conducted for this cluster

CONCLUSION: probable new gene

CLUSTER 1154
sctg_77 : 414499..420533

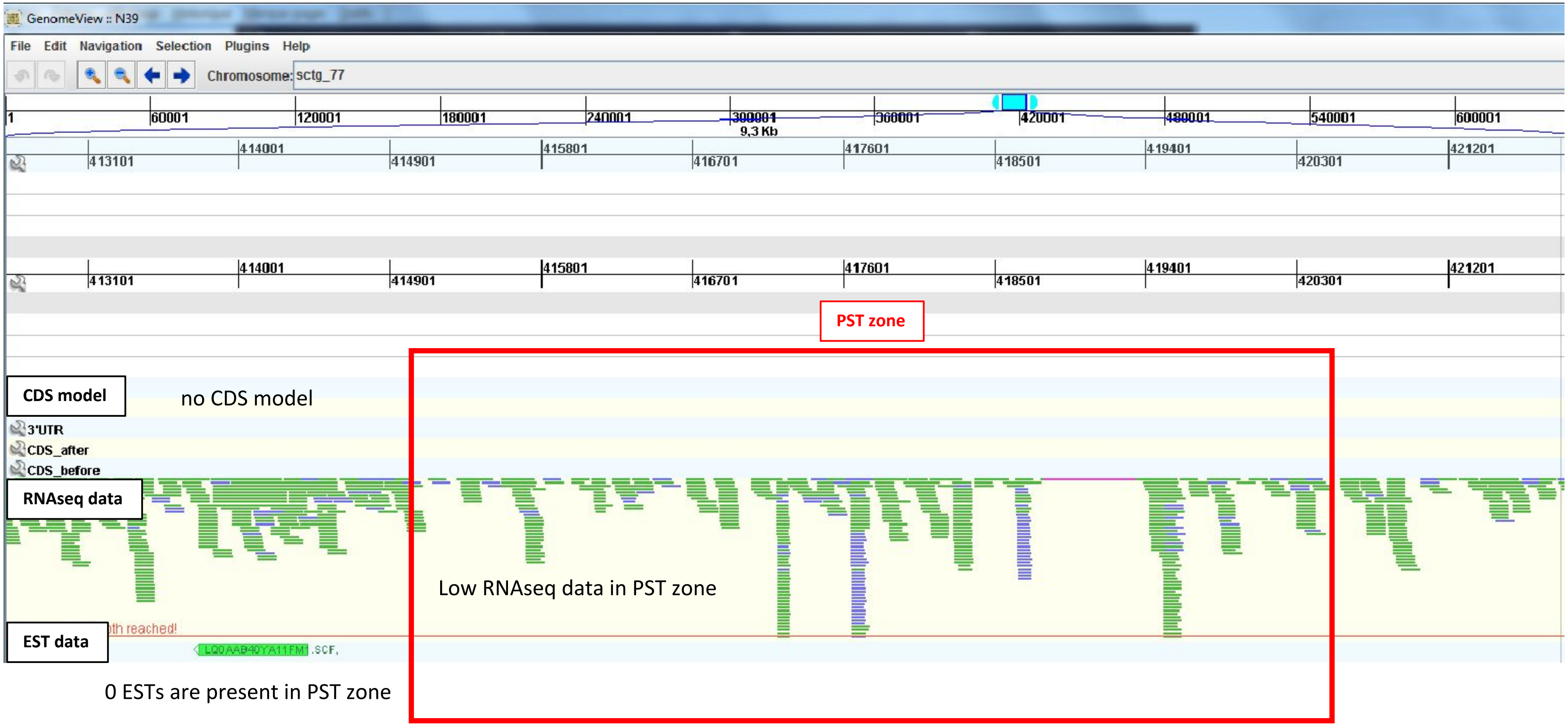

RT-PCR data

No RT-PCR experiment was conducted for this cluster

CONCLUSION: probable new gene

CLUSTER 120
sctg_123 : 77652..82101

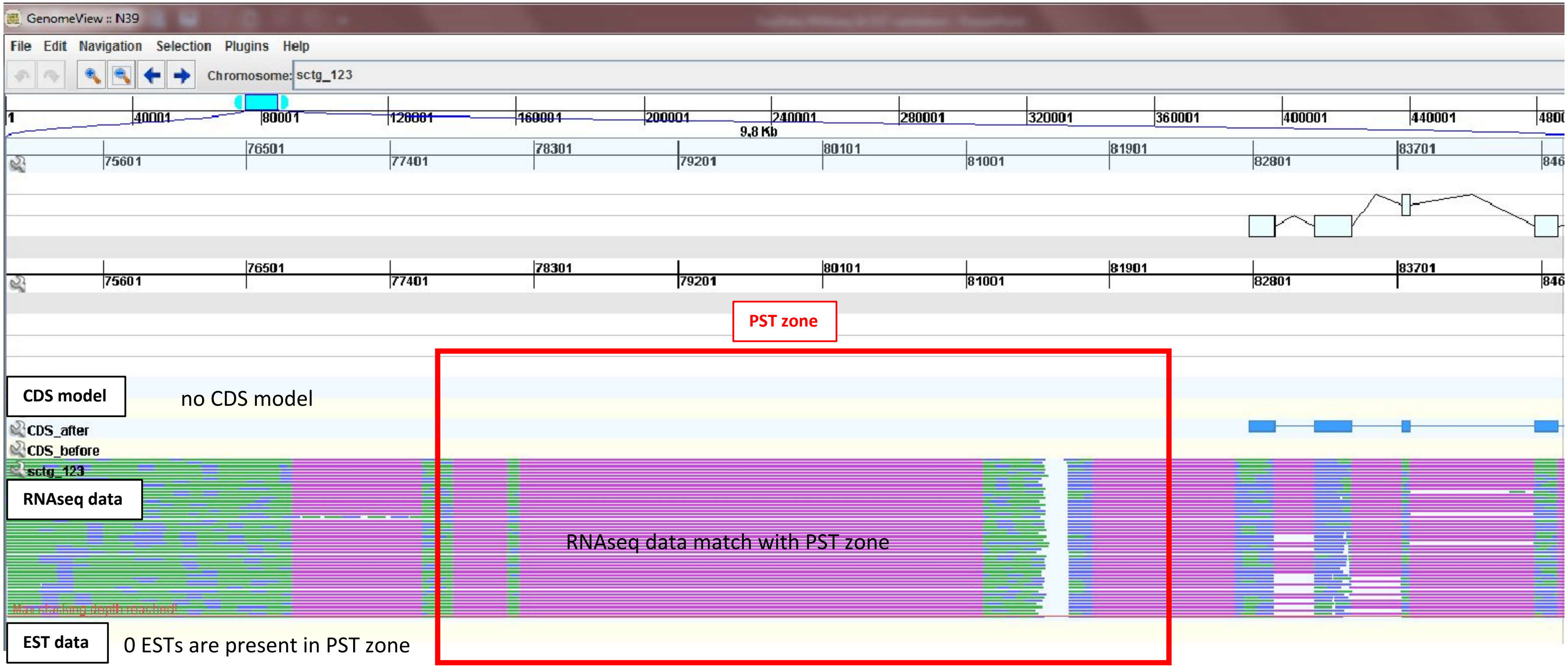

RT-PCR data

No RT-PCR experiment was conducted for this cluster

CONCLUSION: probable new gene

CLUSTER 220
sctg_150 : 399071..404913

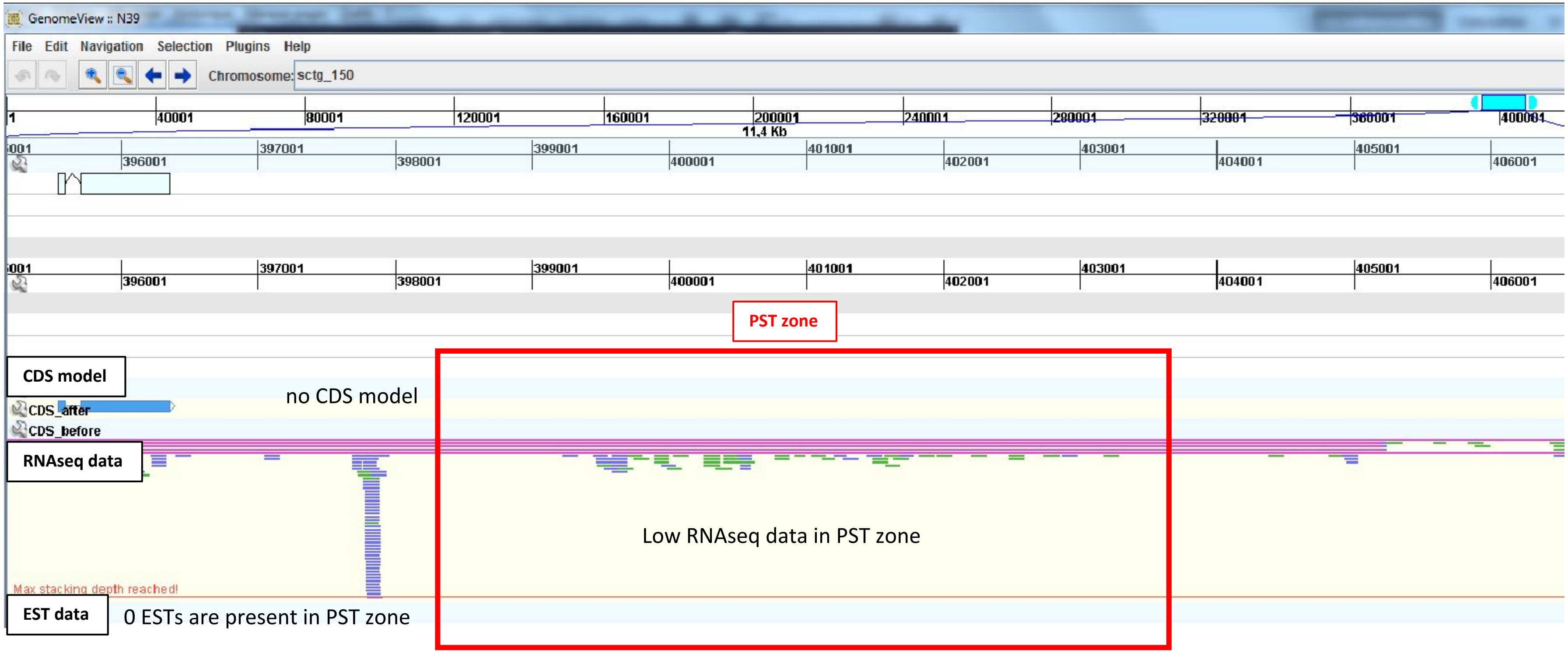

RT-PCR data No RT-PCR experiment was conducted for this cluster

CONCLUSION: probable new gene

CLUSTER 777
sctg_43 : 146476..147692

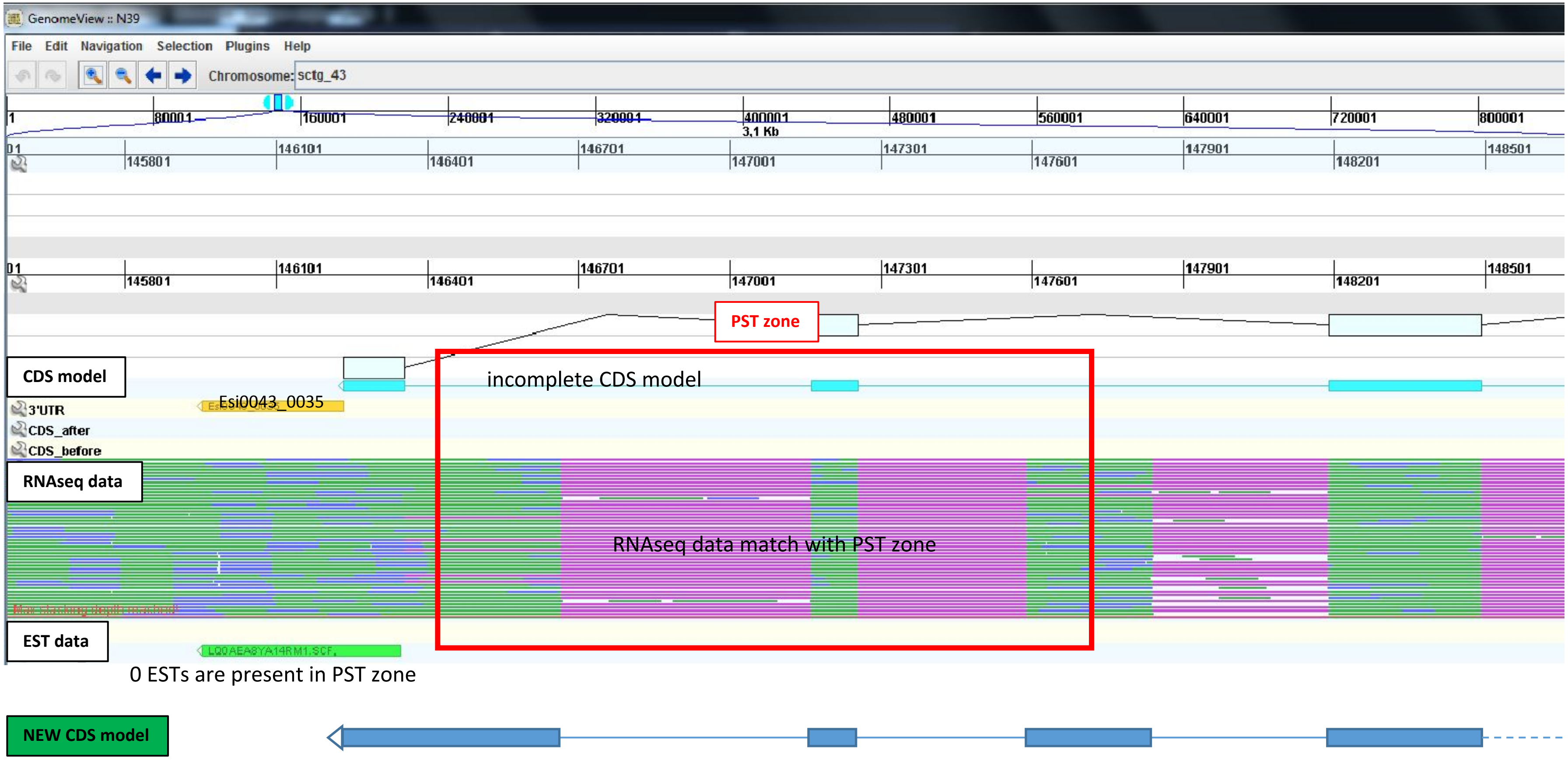

RT-PCR data

No RT-PCR experiment was conducted for this cluster

CONCLUSION: Esi0043_0035 model correction (catalase)
Internal and 3’ end model correction on two exons based on RNAseq data coverage together with the PST zone

CLUSTER 822
sctg_48 : 267223..267911

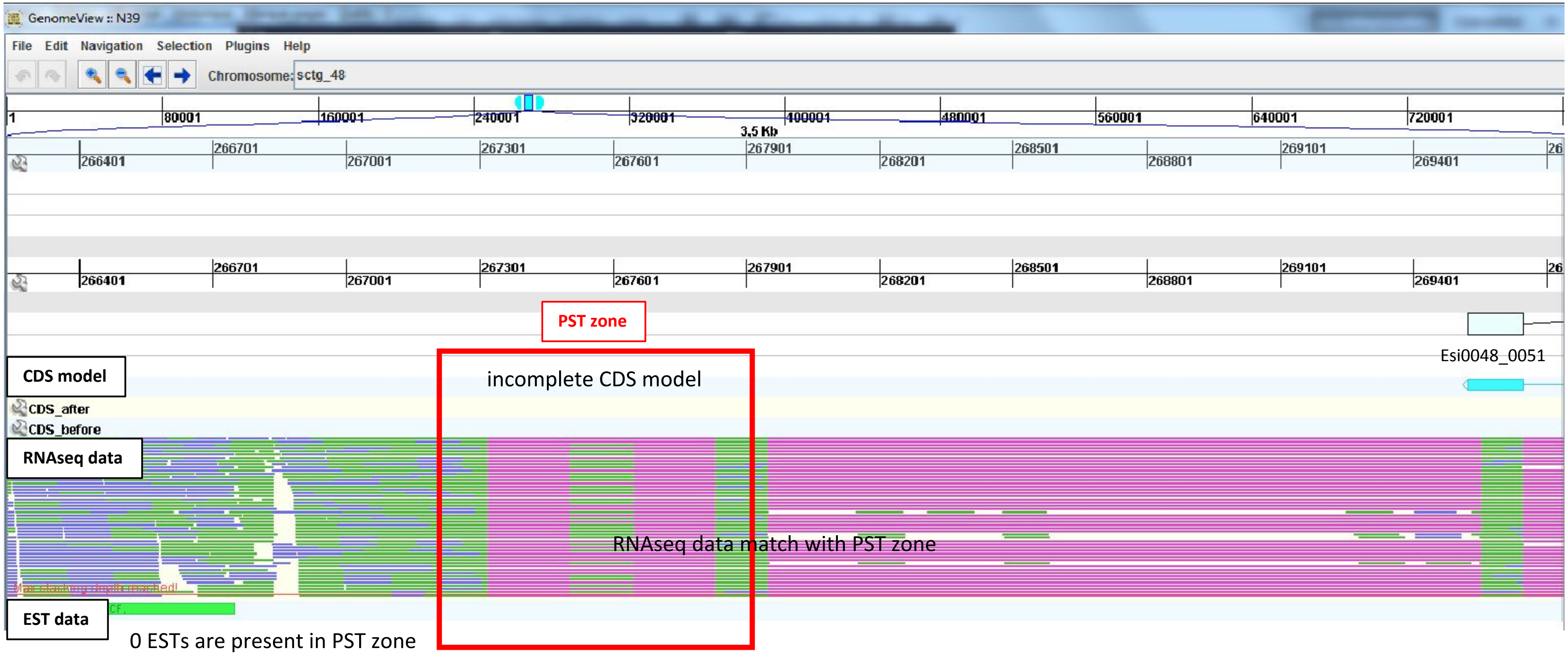

NEW CDS model

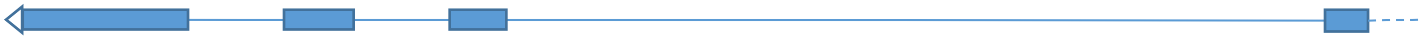

RT-PCR data

No RT-PCR experiment was conducted for this cluster

CONCLUSION: Esi0048_0051 model correction (hypothetical protein)
3' end model correction with 3 supplementary exons based on RNAseq data coverage together with the PST zone

CLUSTER 492

sctg_253 : 215846..216917

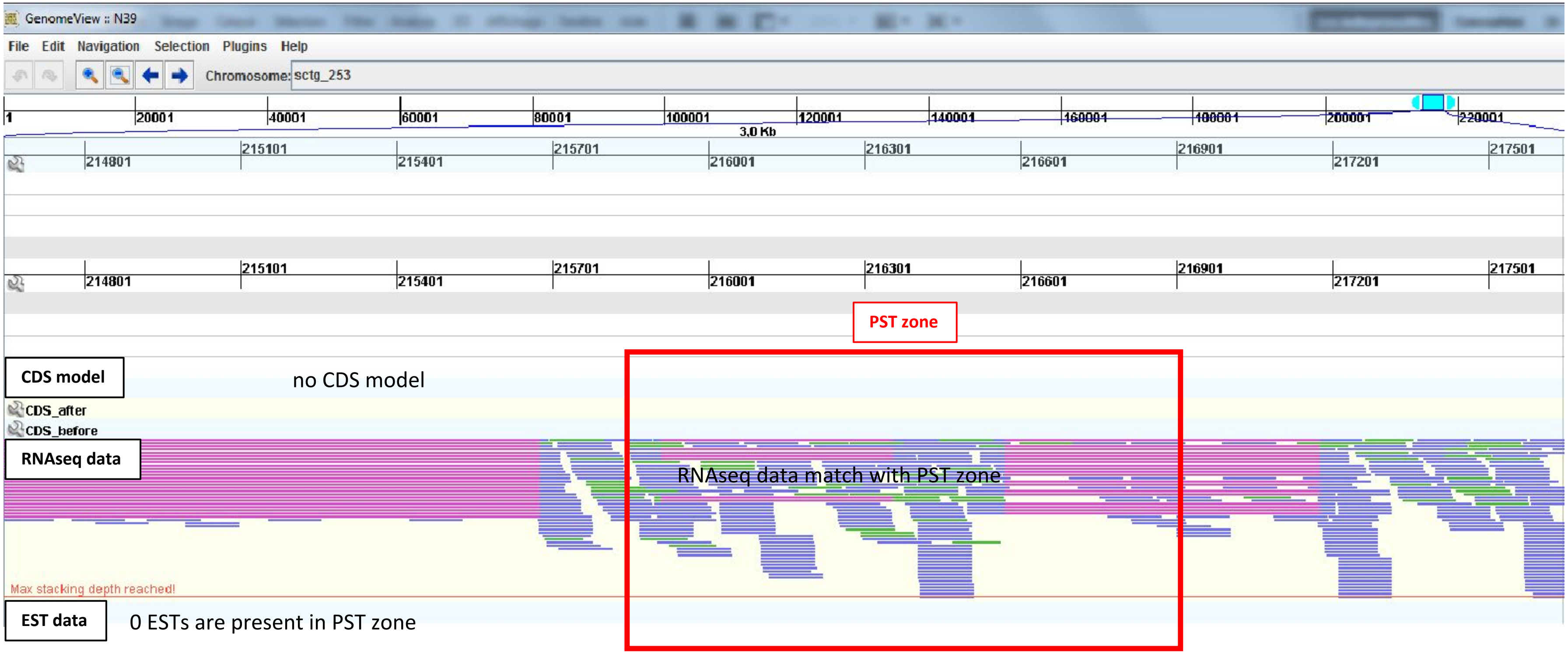

RT-PCR data No RT-PCR experiment was conducted for this cluster

CONCLUSION: probable new gene

CLUSTER 567
sctg_291 : 60813..65620

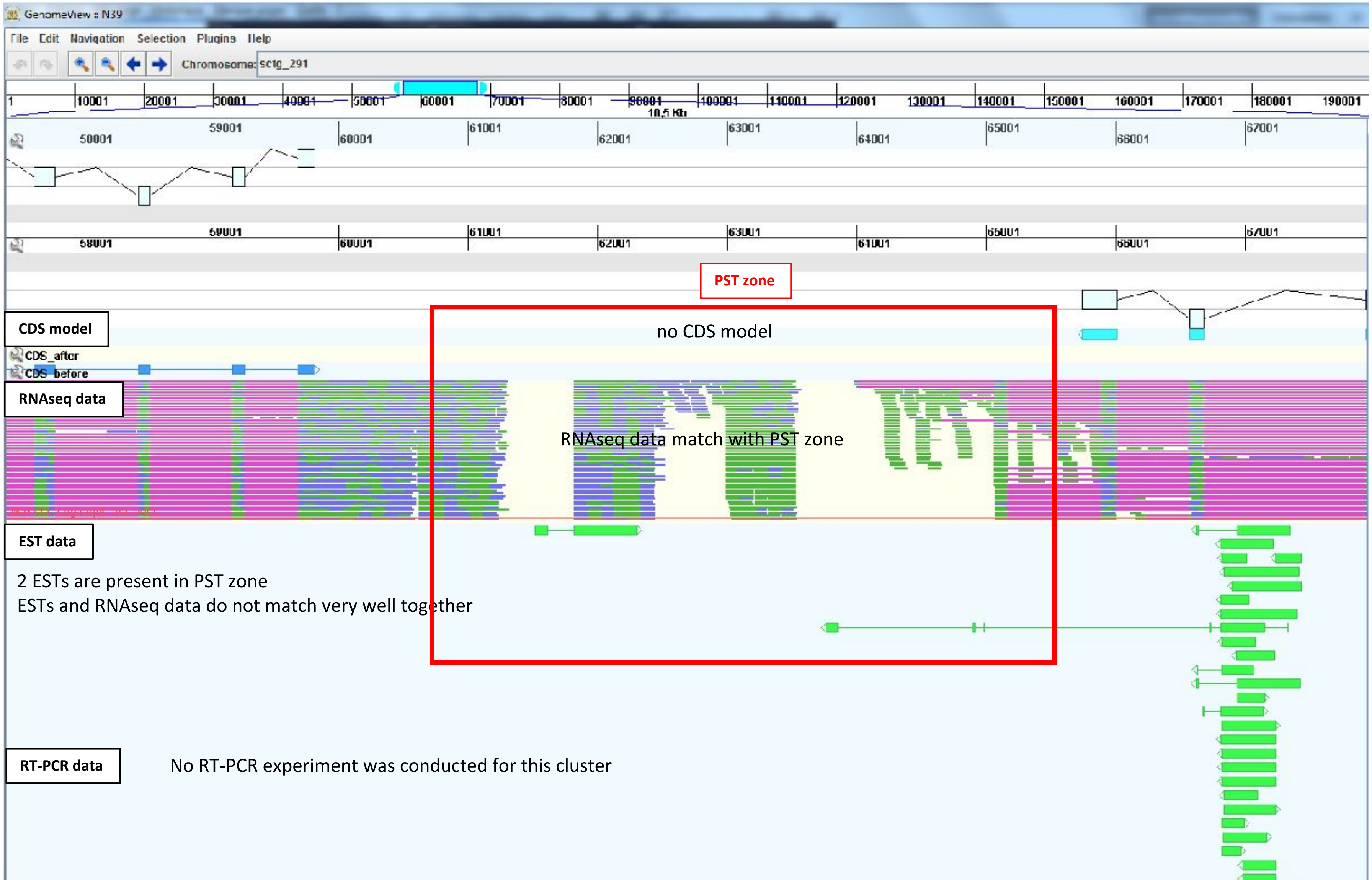

CONCLUSION: Esi0291_0011 model correction (mTERF domaincontaining protein)
3’ end model correction on two exons based on RNAseq data coverage together with the PST zone

CLUSTER 618

sctg_310 : 63974..71773

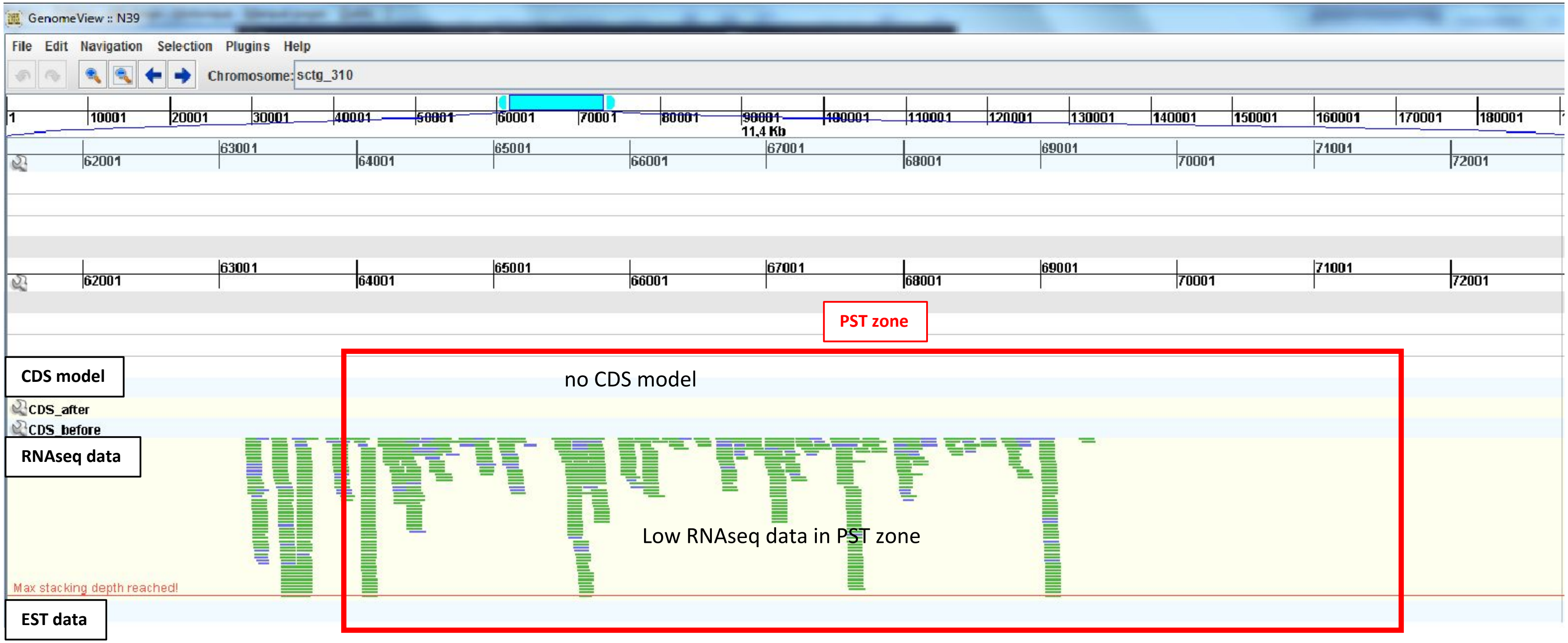

0 ESTs are present in PST zone

RT-PCR data

No RT-PCR experiment was conducted for this cluster

CONCLUSION: probable new gene

CLUSTER 697

sctg_365 : 137829..143627

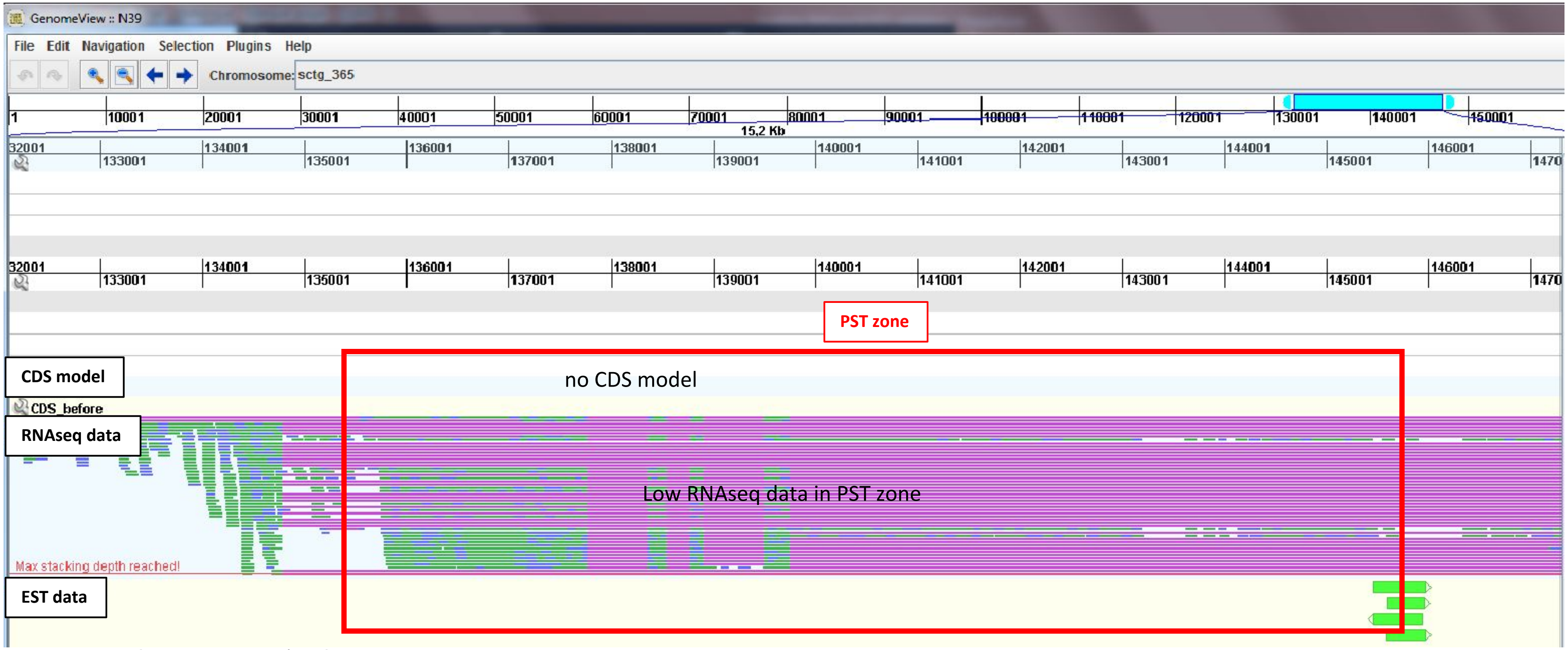

ESTs are present in PST zone
ESTs and RNAseq data do not match together

RT-PCR data No RT-PCR experiment was conducted for this cluster

CONCLUSION: probable new gene

CLUSTER 218
sctg_87 : 471174..478804

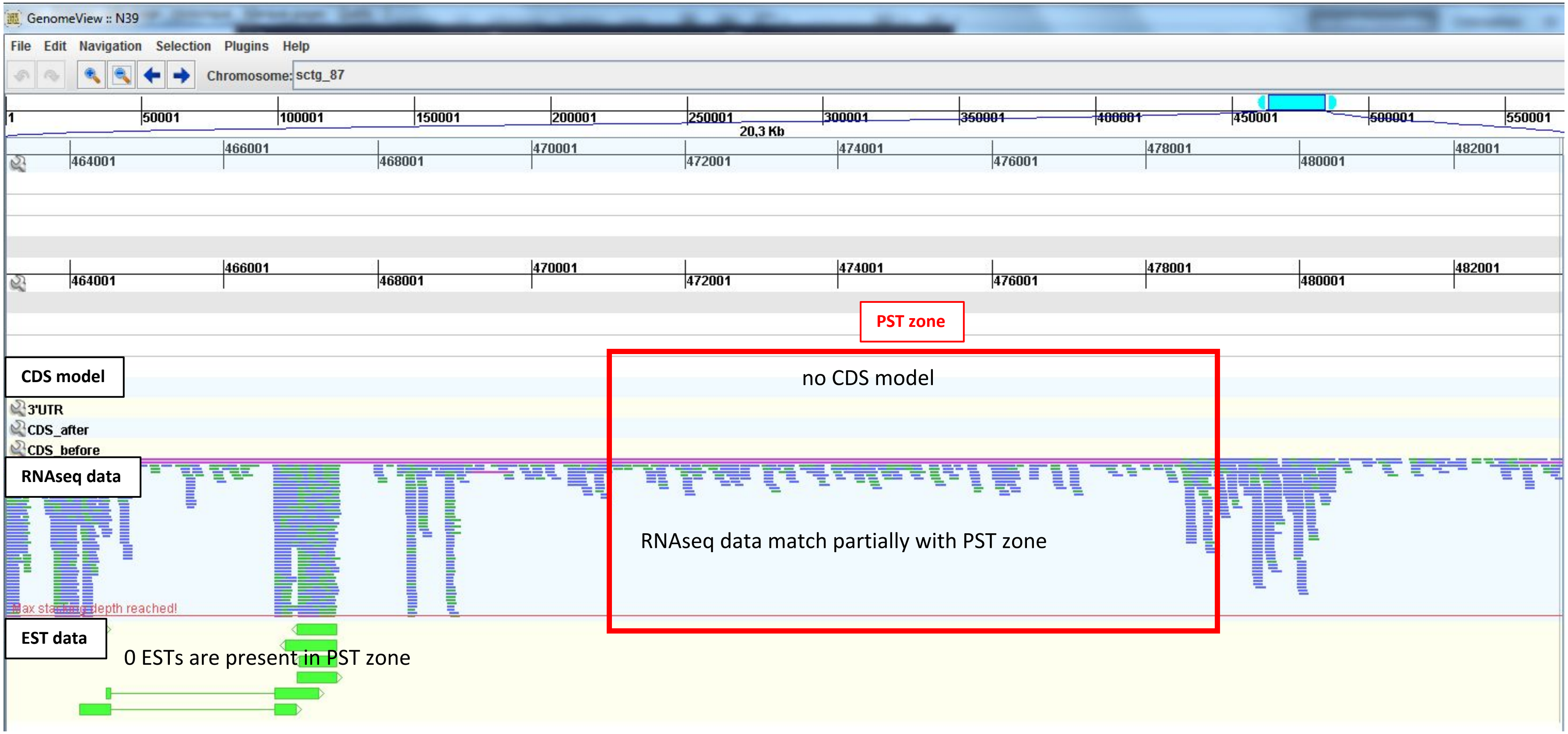

RT-PCR data No RT-PCR experiment was conducted for this cluster

`CONCLUSION: probable new gene (retrotransposon integrase-like protein)`
